# A fully human pan VL9 HLA-E TCRm antibody enables functional dissection of HLA-E biology and checkpoint signaling

**DOI:** 10.1016/j.isci.2026.115669

**Published:** 2026-04-09

**Authors:** Soroush Ghaffari, Katherine Upchurch-Ange, Gizem Oter, Trivendra Tripathi, Susanne Gimlin, Debra Wawro Weidanz, Jim Middelburg, Thorbald van Hall, Jon A. Weidanz

**Affiliations:** 1 Department of Kinesiology, College of Nursing and Health Innovation, The University of Texas at Arlington, Arlington, TX, USA; 2 Department of Bioengineering, College of Engineering, The University of Texas at Arlington, Arlington, TX, USA; 3 Institute of Biomanufacturing and Precision Health for North Texas (IMPRINT), The University of Texas at Arlington, Arlington, TX, USA; 4 Department of Medical Oncology, Oncode Institute, Leiden University Medical Center, Leiden, the Netherlands; 5 Abexxa Biologics, Inc., Arlington, TX, USA; 6 Boehringer Ingelheim Pharmaceuticals, Inc., Forth Worth, TX, USA

**Keywords:** biological sciences, immunology

## Abstract

HLA-E’s function as an immune checkpoint in cancer depends on its display of the canonical peptide (VL9), yet direct profiling of these complexes has been stymied by lack of specific reagents. We now introduce ABX002, a fully human TCR-mimic antibody capable of recognizing all tested VL9/HLA-E complexes with high affinity and specificity *in situ*. Using ABX002, we reveal that canonical VL9/HLA-E surface expression is tightly controlled by inflammatory cues, remarkably infrequent on tumors without stimulation, and almost absent from immune cells except myeloid-lineage cells. ABX002 unlocks cell-type and context-specific quantification of HLA-E antigen presentation, providing unprecedented insight into immune evasion and regulation. It additionally disrupts the NKG2A checkpoint, restoring cytotoxic lymphocyte function and enabling mechanistic and therapeutic mapping of HLA-E restricted peptide presentation. Together, these findings position ABX002 as a transformative tool for dissecting the landscape and biology of canonical peptide restriction in cancer immunity.

## Highlights

• ABX002 is the first fully human TCRm antibody recognizing all tested VL9/HLA-E
• Inflammatory signals are essential for tumor cells to express VL9/HLA-E
• Myeloid cells uniquely display VL9/HLA-E even without inflammatory cues
• ABX002 disrupts the NKG2A checkpoint and unleashes cytotoxic immune cell activity

## Graphical abstract

**Figure:**
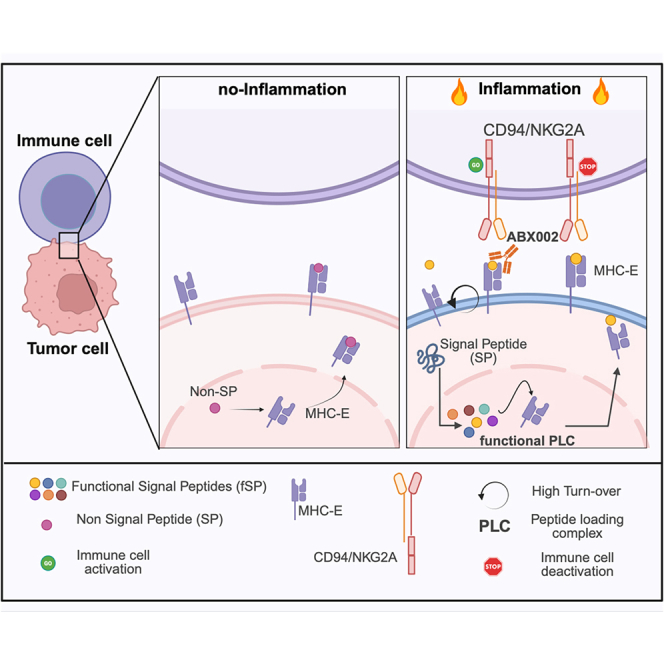

## Introduction

Effector cell dysfunction in cancer is driven in part by immune checkpoint pathways that impair cytotoxic responses.^1^^,^^2^ Among these is NKG2A, a C-type lectin protein that forms a heterodimeric receptor by pairing with CD94 on the surface of effector immune cells, including natural killer (NK) and cytotoxic T lymphocytes (CTLs), to recognize MHC-E, a non-classical MHC class I molecule.^3^^,^^4^^,^^5^^,^^6^ Unlike the highly polymorphic classical MHC class I molecules, MHC-E (HLA-E in humans, Qa-1 in mice, and Mamu-E in rhesus monkeys) is conserved and essentially monomorphic, with two common human allomorphs, HLA-E∗01:01 and HLA-E∗01:03, differing by a single amino acid outside of the peptide binding pocket that does not impact peptide selectivity.^7^^,^^8^^,^^9^ Although typically expressed at low levels in healthy tissues, HLA-E is frequently upregulated in malignancies such as lung,^10^^,^^11^^,^^12^ovarian,^13^^,^^14^ colorectal,^15^^,^^16^^,^^17^^,^^18^ multiple myeloma,^19^ and glioblastoma,^20^ where it correlates with poor prognosis.^21^^,^^22^^,^^23^ In addition, HLA-E undergoes rapid internalization and continuous turnover, making its surface expression highly dynamic and tightly regulated.^24^^,^^25^

HLA-E predominantly presents canonical peptides with the xMAPRTxxL motif, which is derived from signal peptides (SPs) of classical MHC class I^26^ and non-classical HLA-G.^27^^,^^28^ In humans, these peptides are often referred to as VL9 or VMA, while in mice are called Qdm (Qa-1 determinant modifier). Recent work has shown that in humans only a subset of these SPs termed functional SPs (fSPs), are efficiently processed by signal peptidases and loaded onto HLA-E. Only fSPs loaded on HLA-E can promote immune evasion upon engaging with CD94/NKG2A inhibitory receptor.^29^ Lin et al. showed these variations among fSPs directly influence their binding affinity to HLA-E and, subsequently, their ability to induce an inhibitory signal through CD94/NKG2A. For instance, fSPs derived from the HLA-B7 leader sequence were shown to better stabilize the HLA-E complex compared to others while demonstrating reduced NKG2A engagement and signaling.^29^ Additionally, our team^30^ and others^14^^,^^31^ have demonstrated proinflammatory cytokines such as IFN-γ and INF-α can facilitate the loading of fSPs into HLA-E and Qa-1 and increase the density of fSP/MHC-E complexes on the surface of tumor cells, thereby also contributing to immune evasion.

Our team recently characterized EXX-1, a TCRm antibody employed as a mechanistic probe to selectively detect the Qdm/Qa-1^b^ complex on murine tumor and immune cells.^25^^,^^30^ The study demonstrated that the presentation of the Qdm (mouse ortholog of humanVL9) peptide by Qa-1^b^ requires both an intact peptide loading complex (PLC) and an inflammatory stimulus. Surprisingly, Qdm/Qa-1^b^ complexes were observed exclusively on myeloid cells including monocytes, conventional dendritic cells (cDCs), and macrophages despite Qa-1^b^ being broadly expressed across all immune cell types.^25^ This was unexpected, as it is widely assumed that under homeostatic conditions, Qa-1^b^ molecules are constitutively loaded with Qdm peptides and presented on the cell surface. These findings challenge the prevailing view that VL9 peptides like Qdm are commonly presented by Qa-1^b^ across diverse immune and non-immune cells, and they prompt new questions regarding Qa-1^b^ conformers and the nature of peptides presented during both homeostasis and disease. Importantly, the use of a TCRm antibody specific for the Qdm/Qa-1^b^ complex has yielded new insights into Qa-1^b^ biology.

Developing antibodies that specifically recognize HLA-E has proven difficult due to its high sequence homology with classical HLA class I molecules. Widely used antibodies such as 3D12, 4D12, and MEM-E/02 have been instrumental for studying HLA-E expression for over two decades.^32^^,^^33^^,^^34^^,^^35^ However, their broad epitope specificity often results in recognition of peptide-loaded, peptide-free, or even denatured protein. More recently, antibodies like TFL-033^22^^,^^36^ and 3H4^37^^,^^38^ have allowed detection of peptide-free HLA-E or, in the case of 3H4, a specific SP (VMAPRTLLL, L-A1) among six known fSPs, but their specificity for the full spectrum of fSP/HLA-E complexes is limited, restricting utility for mechanistic and therapeutic studies.

Given the established inhibitory role of the fSP/HLA-E:NKG2A interaction in dampening effector cell responses, serveral therapeutic strategies have sought to disrupt this axis. While NKG2A blocking antibodies like monalizumab,^27^^,^^39^ have demonstrated promising pre-clinical results, particularly in combination with PD-1/PD-L1 inhibitors,^40^^,^^41^^,^^42^ there limited efficacy as a monotherapy has prompted alternative strategies, including direct targeting of fSP/HLA-E complex, to overcome tumor-induced immune suppression. However, antibodies like TFL-033 and 3H4 remains constrained by their limited peptide specificity, insufficient for broadly disrupting NKG2A signaling or fully dissecting the biology of HLA-E-mediated antigen presentation. Consequently, there is a critical need for new antibodies that specifically recognize VL9 or broadly fSP-loaded HLA-E.

Here, we describe the development and characterization of ABX002, a fully human TCRm antibody that binds with high affinity to HLA-E presenting any of the six known fSPs. ABX002 enables selective targeting of the fSP/HLA-E complex, disrupting NKG2A-mediated immune suppression while preserving other physiological functions of HLA-E. Importantly, ABX002 serves as a molecular probe to investigate the biology of VL9 peptide presentation, HLA-E trafficking, and immune checkpoint regulation. *In vitro*, ABX002 enhances NK and CD8^+^ T cell mediated cytotoxicity against fSP/HLA-E^+^ tumor cells. *In vivo*, a surrogate antibody^30^ targeting Qdm/Qa-1^b^ the murine ortholog of VL9/HLA-E recapitulates ABX002’s activity, reducing tumor burden and synergizing with PD-(L)1 blockade. Together, these findings establish ABX002 as both a mechanistic tool and a therapeutic agent, offering new insights into HLA-E biology and a unique strategy to overcome immune suppression in cancer.

## Results

### Introduction of peptides used and nomenclature

A list of peptide sequences with gene origin is provided in Table 1. The panel of canonical SPs used for selecting the ABX002 antibody includes functional (fSPs) and non-fSPs. Amino acid residues that differ between the canonical SPs relative to the L-C3 peptide are shown in red. SPs were labeled according to their HLA class I allele of origin (e.g., L-B7 for B∗07:02, B∗07:05, etc. or L-G for G∗01, G∗02, etc.). Additionally, control peptides used for determining specificity include non-canonical self peptides and pathogen-derived peptides known to form stable complexes with HLA-E.

**Table 1:** The study peptides used with their gene of origin

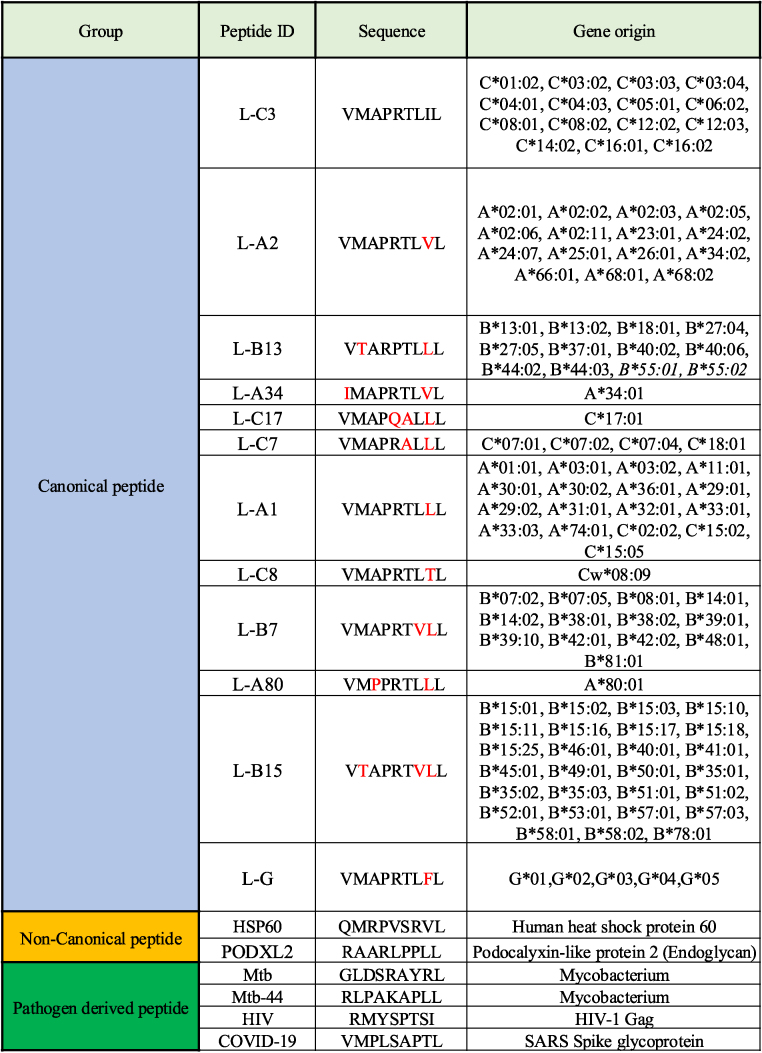

### Selection of a scFv clone specific for fSP/HLA-E

A single phage clone specific for L-G/HLA-E (Table 1) was identified from a human semi-synthetic naive library and reformatted for yeast display as a scFv. The parental clone exhibited modest binding affinity (K_D = 410 nM), which was improved by more than 10-fold through yeast-based affinity maturation. Following iterative rounds of selection, 12 unique scFv clones were identified, each displaying a 10- to 100-fold increase in binding affinity relative to the parental clone. One clone was selected based on its binding to L-G/HLA-E at 1 and 0.1 nM concentrations (Figure S1A), designated ABX002, and reformatted as a full-length antibody for subsequent characterization.^43^

### Characterization of ABX002

**Figure 1:**
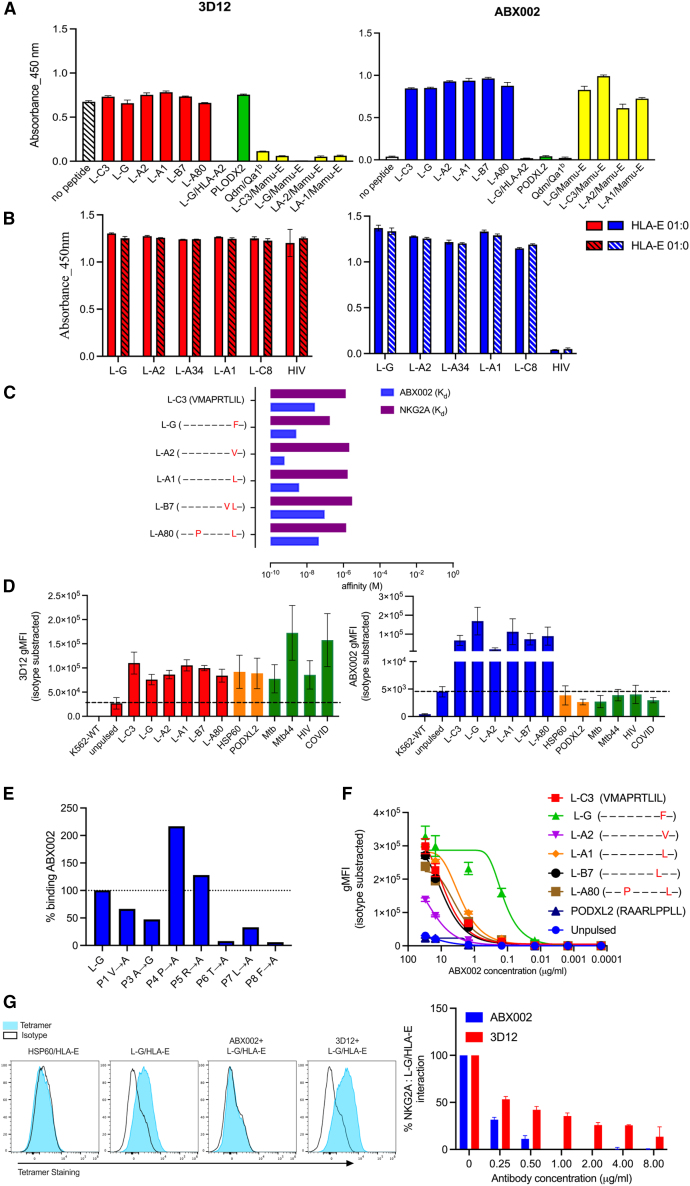
ABX002 exhibits peptide selectivity for fSP/HLA-E complexes (A) ELISA-based binding of ABX002 and 3D12 to peptide/HLA-E complexes. Left: 3D12; right: ABX002 binding to a panel of peptides, including fSPs and control peptide HLA-E complexes. Red and blue indicate fSP/HLA-E detection by 3D12 and ABX002, respectively; green denotes detection of non-canonical peptides or non-SPs presented by HLA-E; yellow indicates detection of fSP/HLA-E orthologs from mouse or non-human primates. (B) ELISA comparing ABX002 and 3D12 binding to HLA-E isoforms. Left: 3D12; right: ABX002 binding to SPs or control peptide presented by either HLA-E01:03 or HLA-E∗01:01. (C) Affinity measurements of ABX002 for all fSP/HLA-E complexes compared to NKG2A. Binding kinetics was assessed using ResoSens Ultra Mab Pro. (D) Cell-based analysis of ABX002 binding to peptide-loaded K562 cells expressing HLA-E (K562-E). K562-E cells were pulsed with fSPs or control peptides and stained with 3D12 (red) or ABX002 (blue). Orange indicates non-canonical peptides, and green represents pathogen-derived peptides. Dashed line represents the basal detection level of 3D12 in unpulsed cells or of ABX002 in the absence of pulsed peptide. (E) Alanine scanning of the L-G fSP in K562-E cells. ABX002 binding is quantified as gMFI of the indicated peptide normalized to gMFI of L-G × 100. Dashed line indicates ABX002 binding to cells pulsed with wild-type peptide, used as a reference for comparing other pulsed conditions. (F) Titration of ABX002 on K562-E cells loaded with fSPs or control peptides. (G) HLA-E tetramer staining of NKG2A^+^ NK-92 cells. L-G/HLA-E tetramer staining was assessed in the presence or absence of HLA-E blockade using 3D12 or ABX002 to disrupt the NKG2A axis. HSP60/HLA-E tetramer used as control protein for this assay. Data are represented as mean ± SD of *n* = 3. See also Figure S1.

The selectivity of ABX002 was first analyzed by ELISA using an array of SP/MHC-E complexes. As shown in Figure 1A, ABX002 recognized all tested SP peptides, including all the fSP presented by HLA-E (blue), and did not bind to HLA-E without a peptide, HLA-E with control peptides, HLA-A2 with an L-G peptide or the mouse ortholog (Qdm/Qa-1^b^). Importantly, it did recognize fSP peptides presented in MAMU-E, the Rhesus macaque (RM) ortholog of HLA-E (Figure 1A). Meanwhile, commercial anti-HLA-E clone 3D12 recognized all HLA-E complexes tested, although it did not recognize Qdm/Qa-1^b^ nor MAMU-E, as has been reported.^32^ ABX002 and 3D12 binding profile to non-fSPs (additional SP peptides) presented by HLA-E is shown in Figure S1B. ABX002 also recognized SP peptides in both HLA-E alleles (HLA-E∗01:01 and HLA-E∗01:03), as did 3D12 (Figure 1B) while no binding was detected to HLA-E allesles loaded with control peptide (HIV). The binding affinity of ABX002 to all fSP variants was determined and compared with previously reported binding affinity of fSPs to NKG2A/C.^29^ Affinities and dissociation equilibrium constant (K_D_) of ABX002 to its targets ranged from 0.597 nM against L-A2/HLA-E to 27.8 nM against L-C3/HLA-E, compared with affinities of NKG2A to fSP/HLA-E ranging from 180 μM against L-G/HLA-E to 2.98 mM against L-B7/HLA-E (Figure 1C). This indicates an average difference in affinity of 5.5 logs for fSP/HLA-E with ABX002 binding markedly stronger than NKG2A. A list of ABX002 and NKG2A/C binding affinities to fSP/HLA-E is given in Figure S1C. Binding selectivity of ABX002 was further demonstrated using peptide-pulsed K562 cells transfected with HLA-E (K562-E). Binding to peptide-pulsed cells closely mirrored binding measured via ELISA, with ABX002 only recognizing fSP/HLA-E peptides while 3D12 was not peptide-selective (Figure 1D). Low levels of binding to unpulsed K562-E were observed, and this can be explained by endogenous intracellular expression of HLA-A11, HLA-B18, and HLA-C5 in K562 cells.^44^ ABX002 binding to additional SP peptides (non-fSP) was also confirmed in peptide-pulsed K562-E (Figure S1D). To assess epitope binding of ABX002, seven L-G peptide variants containing single alanine or glycine substitutions (A1, G3, A4, A5, A6, A7, and A8) were synthesized and used to pulse K562-E cells. Substitutions at positions p2 and p9 were excluded because these are primary anchor residues for L-G binding to HLA-E. As shown in Figure 1E, ABX002 staining revealed broad interactions across the L-G peptide, with a marked reduction (75%–95%) in binding to peptides G3, A6, A7, or A8 compared with wild-type L-G. In contrast, substitutions at positions 4 and 5 enhanced ABX002 binding, indicating that these residues modulate antibody recognition rather than constituting critical contact sites. Collectively, these data demonstrate that ABX002 relies on key interactions with residues 3, 6, 7, and 8 of the L-G peptide. To validate the presence of the mutated peptide in the HLA-E binding pocket, the 3D12 antibody was used as a control in this assay (Figure S1E). ABX002 binding avidity for all fSP was demonstrated through binding titration assays conducted on fSP-pulsed K562-E cells (Figure 1F). The selectivity of ABX002 to fSP was additionally shown through a competition assay using NK-92 cells and L-G/HLA-E tetramer. L-G/HLA-E tetramer bound to NK2GA on the surface of NK-92 cells and this interaction was blocked entirely by the addition of 1.0 μg/mL of ABX002. 3D12 antibody was used as control antibody for this assay and results indicated partial inhibition of L/G/HLA-E tetramers to NKG2A/CD94 at 8.0 μg/mL (Figure 1G). Gating strategies of the blocking assay with ABX002 or 3D12 are shown in Figure S1F.

### ABX002 binding in tumor cell lines requires the presence of fSP induced by inflammatory signal

**Figure 2:**
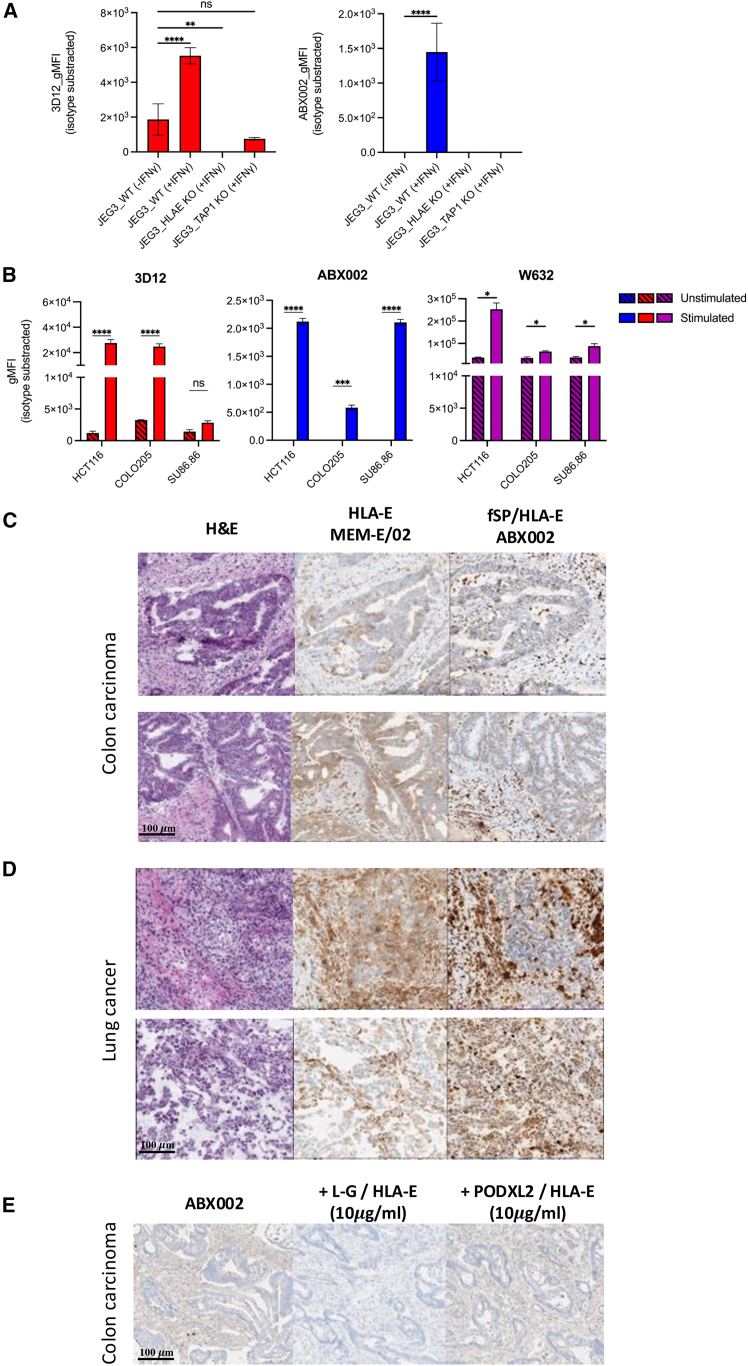
ABX002 selectively binds tumor cell lines and tissue samples presenting fSP/HLA-E complexes Cell lines and tissue sections were stained with the indicated antibodies to assess the binding specificity of ABX002. (A) Flow cytometry analysis of wild-type (WT) JEG3 cells with or without IFN-γ stimulation, and IFN-γ stimulated, genetically modified JEG3 cells with HLA-E or TAP-1 knockout. Cells were stained with ABX002 (blue) or 3D12 (red) to assess fSP/HLA-E complex presentation. (B) Staining of various tumor cell lines expressing classical MHC class I, HLA-E, or fSP/HLA-E complexes. Detection was performed using W632 (purple), 3D12 (red), or ABX002 (blue), in the presence or absence of IFN-γ stimulation. (C and D) Histopathological analysis of fresh frozen (C) colon carcinoma and (D) lung cancer tissue sections. Hematoxylin and eosin (H&E) staining was performed to assess tissue architecture. Adjacent sections were stained with ABX002 to detect fSP/HLA-E complexes and with anti-HLA-E antibody (clone MEM-E/02) to detect total HLA-E protein. Staining with MEM-E/02 and corresponding isotype control was visualized using the Ventana Discovery Ultra Platform and the Discovery OmniMap anti-Mouse HRP Kit. ABX002/mIgG1 and its isotype control were detected using the Ventana Discovery Amp HQ Kit and Discovery Amplification anti-HQ-HRP Multimer. Scale bars, 100 μm. (E) Competitive inhibition of ABX002 binding to colon carcinoma tissue in the presence of L-G/HLA-E complexes or control peptide (PODXL2) loaded HLA-E. Data are represented as mean ± SD of *n* = 3 (A, B). Statistical analysis was performed using one-way ANOVA for (A) and Student’s *t* test for (B). ∗*p* < 0.05, ∗∗*p* < 0.01, ∗∗∗*p* < 0.001, and ∗∗∗∗*p* < 0.0001. See also Figure S2.

Selectivity of ABX002 was demonstrated by knocking out genes encoding for *HLA-E* or the transporter associated with antigen processing 1 (*TAP1*) in JEG3. As shown in Figure 2A, IFN-γ stimulated wild-type JEG3 cells to exclusively present fSP/HLA-E allowing ABX002 binding. However, ABX002 binding was completely abrogated when *HLA-E* gene was knocked out. Similarly, ABX002 binding was not observed in *TAP1* knock out cells indicating TAP1 protein dependence for transporting classical and non-classical MHC class I SPs into the endoplasmic reticulum for loading into HLA-E. 3D12 antibody staining confirmed the successful knock out of HLA-E and further demonstrated that TAP1 disruption impacts HLA-E surface expression. To further validate the binding selectivity of ABX002 for HLA-E presenting fSP, several tumor cell lines were stained using clone 3D12 for HLA-E expression, W6/32 for class I HLA and ABX002. Cell lines were either left unstimulated or treated overnight with IFN-γ to upregulate HLA-E expression and to promote fSP peptide loading into HLA-E (Figure 2B, left). ABX002 binding was only observed in cell lines after IFN-γ treatment (Figure 2B, center). Notably, ABX002 binding did not correlate with W6/32 binding, as HLA class I levels remained high across all cell lines tested (Figure 2B, right). HLA genotype for tumor cell lines used in this manuscript is provided in Figure S2A. Specific antibody-binding capacity (SABC) measurements using ABX002 demonstrated that endogenous fSP/HLA-E is detectable on tumor cell lines following overnight IFN-γ stimulation, but expressed at very low densities. Quantified ABX002 binding levels were ∼180 molecules per COLO205 cell, ∼264 per HCT116 cell, and ∼355 per JEG3 cell—values that fall below the lower limit of the assay’s standard curve (1,700 antibody molecules per bead) (Figure S2B). By contrast, clone 3D12 exhibited markedly higher binding under identical conditions (COLO205: 24,856; HCT116: 15,184; JEG3: 5,176 3D12 molecules/cell). These results are consistent with prior evidence that fSP/HLA-E is intrinsically low-abundance, typically present at well under ∼1,700 copies per cell.

Histology staining in colon carcinoma (Figure 2C) and lung cancer (Figure 2D) demonstrated ABX002 binding, indicating the presence of the fSP/HLAE complexes in tumor tissue. To further differentiate fSP/HLA-E from total HLA-E protein expression, staining was performed using MEM-E/02 which detects denatured heavy chain of HLA-E and therefore corresponds to total HLA-E protein level expression. Our data revealed similar staining patterns in tumor cells and tumor stroma for ABX002 and MEM-E/02 with ABX002 showing markedly stronger staining of tumor stroma compared to tumor cells. ABX002 binding in tumor tissue was confirmed to be specific to fSP/HLA-E, as evidenced by its complete abrogation upon preincubation with an fSP/HLA-E complex (L-G/HLA-E) but not with an irrelevant HLA-E peptide-loaded complex (PODXL2/HLA-E) serving as a control. As shown in Figure 2E, blocking the target complex effectively eliminated ABX002 staining, further validating its specificity for fSP/HLA-E in tumor tissue.

### ABX002 only binds monocytes within leukocyte populations

**Figure 3:**
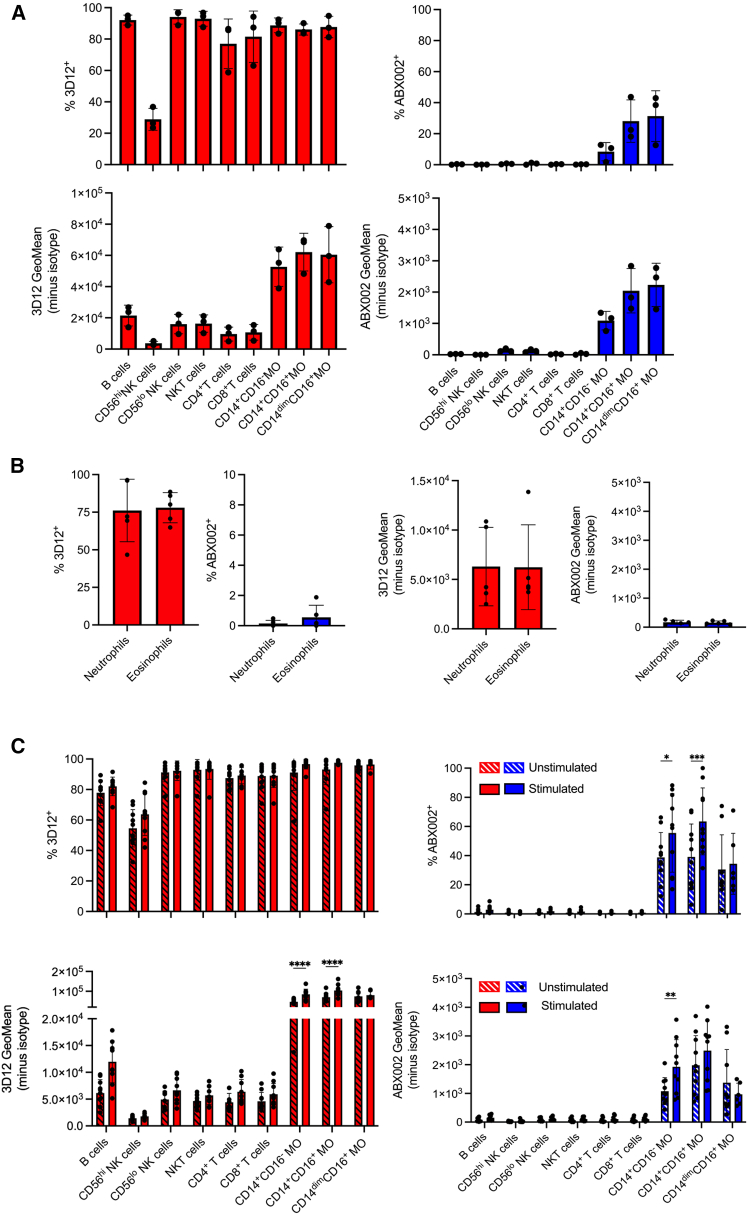
Characterization of fSP/HLA-E expression in normal human PBMCs Semi-quantitative analysis of total HLA-E protein (red, detected by 3D12) and fSP/HLA-E complexes (blue, detected by ABX002) in healthy donor PBMCs. (A) Percent positivity (top) and geometric mean fluorescence intensity (gMFI; bottom) of freshly isolated PBMC subsets stained immediately following isolation (*n* = 3 donors). (B) Characterization of purified neutrophils and eosinophils from healthy donors stained with 3D12 (red) and ABX002 (blue). Left: percent positive cells; right: gMFI (*n* = 4 donors). (C) PBMCs from additional healthy donors were treated overnight with IFN-γ or left un-treated and stained with ABX002 (blue) and 3D12 (red) to assess total HLA-E expression and fSP/HLA-E presentation (*n* = 10 donors). Data are represented as mean ± SD. Statistical significance was determined by one-way ANOVA with Šidák’s multiple comparisons test: *p* < 0.05 (∗), *p* < 0.01 (∗∗), *p* < 0.002 (∗∗∗), and *p* < 0.0001 (∗∗∗∗). See also Figure S3.

Given that peripheral blood mononuclear cells (PBMCs) are known to express HLA-E, we evaluated ABX002 binding to fSP/HLA-E and compared it with total HLA-E protein levels detected by the pan-HLA-E antibody 3D12. The prevailing model in the HLA-E field holds that, under physiological conditions, all nucleated cells including immune cells constitutively present SPs (fSPs) in complex with HLA-E to protect against NK cell mediated lysis by promoting self-tolerance through inhibitory NKG2A signaling. Accordingly, we next assessed ABX002 binding across immune cell subsets as a safety and selectivity evaluation, since broad immune cell recognition could result in unacceptable toxicity. Our initial assessments with fresh PBMC staining revealed robust HLA-E expression across all cell populations tested, with the exception of CD56^hi^ NK cells, of which approximately 30% stained positive for 3D12 (Figure 3A, left images). In contrast, ABX002 binding was restricted to monocytes, with classical monocytes (CD14^+^CD16^−^) exhibiting the lowest staining, and transitional (CD14^+^CD16^+^) and non-classical (CD14^dim^CD16^+^) monocytes displaying progressively higher levels of ABX002 binding (Figure 3A, right images). Notably, granulocytes (neutrophils and eosinophils) also demonstrated high levels of HLA-E (3D12+), yet showed no detectable ABX002 binding (Figure 3B). PBMCs were then treated with IFN-γ or left untreated for 24 h prior to staining, to assess the impact of inflammatory stimulation. While the percentage of HLA-E positive cells was already near maximal across most immune subsets, IFN-γ stimulation further increased HLA-E expression intensity (geometric mean) (Figure 3C, left images). Despite this upregulation, ABX002 binding remained selectively restricted to monocytes regardless of stimulation status (Figure 3C, right images). Moreover, ABX002 binding to monocytes was independent of donor HLA genotype and did not display preferential recognition of specific fSP variants (Figure S3A). The absence of ABX002 binding to other PBMC subsets may reflect low surface copy numbers of fSP/HLA-E complexes below the detection threshold of ABX002, or alternatively, expression of peptide-free HLA-E or HLA-E presenting non-canonical peptides. Representative flow cytometric histograms from a single donor are shown in Figure S3E to illustrate staining intensity and binding specificity. These data demonstrate robust 3D12 staining across immune cell subsets, whereas ABX002 binding is selectively observed on monocytes, enabling direct comparison of total HLA-E expression and ABX002 reactivity. Corresponding flow cytometry gating strategies are shown in Figures S3B and S3C.

### ABX002 poteniates effector cell-mediated killing of tumor cells *in vitro*

**Figure 4:**
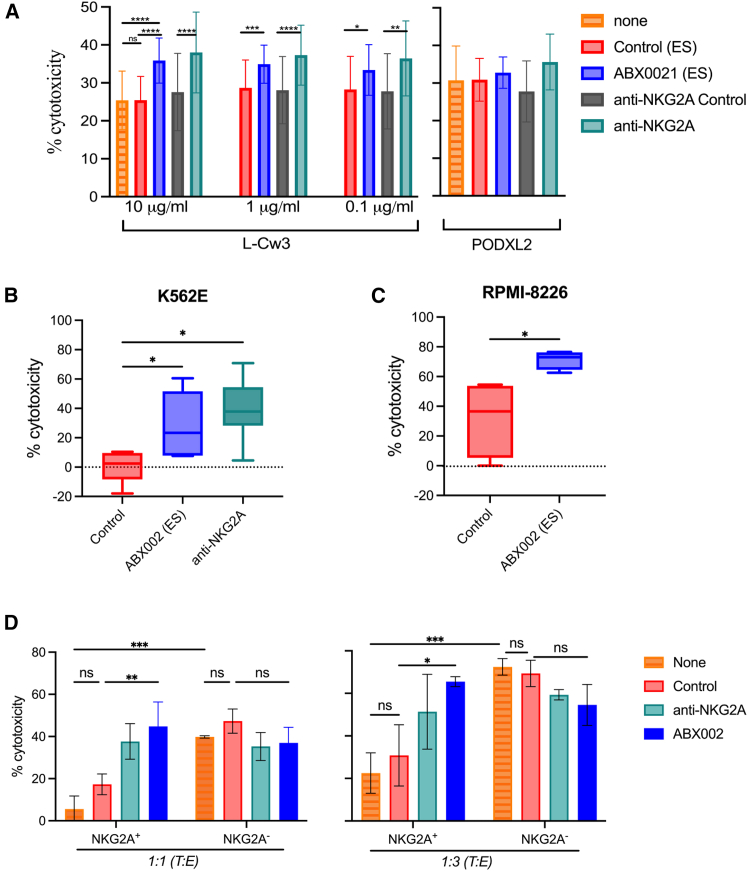
ABX002 unleashes NK- and CTL-mediated cytotoxicity against target cells (A) NK cell mediated cytotoxicity using primary NK cells against IFN-γ treated JY cells pulsed with L-C3 or PODXL2 peptides in the presence of ABX002 (effector-silent format, ES), anti-NKG2A, or corresponding control antibodies. (B) Cytotoxic responses of primary NK cells against K562-E cells positively sorted for fSP/HLA-E complexes, assessed in the presence of ABX002 (ES), anti NKG2A, or control antibodies. (C) NK cell mediated cytotoxicity against RPMI-8226 tumor cells by primary NK cells in the presence of ABX002 (ES) or control antibody. (D) CTL-mediated cytotoxicity by NKG2A^+^ and NKG2A^−^ Flu/M1-specific CD8^+^ T cells against IFN-γ treated JY cells pulsed with M1 peptide, in the presence of ABX002, anti-NKG2A, or controls. Cytotoxicity was assessed at 1:1 (left) and 1:3 (right) target to effector (T:E) cell ratios. Data are represented as mean ± SD. Sample sizes: (A) *n* = 5, (B) *n* = 6, (C) *n* = 4, (D) *n* = 2. Statistical analysis was performed using one-way ANOVA (A, B, D), unpaired *t* test (C). Significance thresholds: *p* < 0.05 (∗), *p* < 0.01 (∗∗), *p* < 0.001 (∗∗∗), *p* < 0.0001 (∗∗∗∗). Error bars represent mean ± SD. Sample sizes: (A) *n* = 5, (B) *n* = 6, (C) *n* = 4, (D) *n* = 2, and (E) *n* = 10. See also Figure S4.

Since ABX002 exhibited highly selective binding in cancer cell lines, its ability to block and disrupt the HLA-E:NKG2A inhibitory axis was assessed in cytotoxicity assays using primary NK cell and fSP peptide pulsed JY cells. In order to prevent any Fc receptor (FcR) mediated effector function, ABX002 was generated with an effector-silent Fc (ABX002 ES). As shown in Figure 4A, NK lysis was enhanced by ABX002 but only when pulsing with a fSP (L-C3). This effect was not seen when JY cells were pulsed with a control (PODXL2) peptide. To further validate these findings, K562-E cells were sorted for binding to ABX002. Double sorted K562-E/fSP+ cells subsequently co-cultured with primary NK cells. After 4 h of co-culture, it was shown that ABX002 significantly augmented NK cell-mediated lysis similar to cultures treated with anti-NKG2A antibody blockade (Figure 4B). Parallel findings were observed when using RPMI-8226 cells where ABX002 treatment compared to control antibody was able to markedly enhance tumor lysis by NK cells (Figure 4C). These results indicate that ABX002 can disrupt the HLA-E:NKG2A axis and unleash NK cell cytotoxicity.

We next investigated ABX002’s impact on NKG2A^+^ and NKG2A^−^ CD8^+^ T cells. CTL lines specific for the influenza M1 peptide (GILGFVFTL) were generated by co-culture with irradiated HLA-A2^+^ PBMCs pulsed with M1 peptide, followed by sorting into NKG2A^+^ and NKG2A^−^ CD8^+^ populations. Sorted CTLs were assessed for cytolytic activity against IFN-γ treated, M1 peptide pulsed JY cells (Figure 4D) and COLO205 cells (Figure S4A) in the presence of control antibody, ABX002, or anti-NKG2A antibody. IFN-γ treatment induced surface expression of fSP/HLA-E complexes on both target cell types. Results from these studies revealed that ABX002 significantly enhanced cytotoxicity of NKG2A^+^ CTLs compared with control conditions or NKG2A^−^ CTLs. Collectively, these findings underscore the potential of ABX002 (ES) as an immune checkpoint inhibitor that can successfully restore NKG2A^+^ CD8^+^ T cells and NK cells lysis of cells. Gating strategies used to select NKG2A ^and^ NKG2A^−^ CTLs are provided in Figure S4C.

### ABX002 surrogate enhances tumor regression and promotes anti-tumor immunity in A20 tumor-bearing BALB/c mice

**Figure 5:**
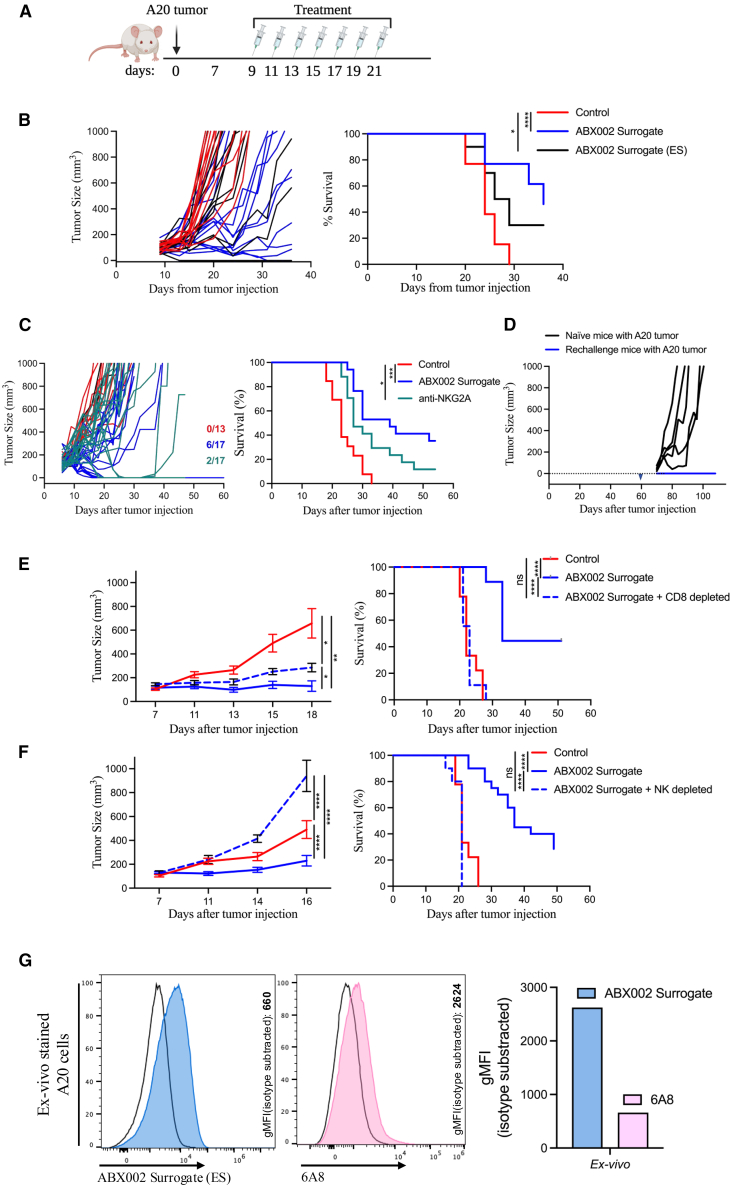
Treatment with the ABX002 surrogate promotes anti-tumor immunity *in vivo* (A) Experimental design schematic for the A20 tumor model evaluating ABX002 surrogate as a therapeutic agent. (B) Individual tumor growth profiles (left) and Kaplan-Meier survival curves (right) of mice treated with two format of ABX002 surrogate or control antibody (*n* = 10 per group). (C) Individual tumor growth profiles (left) and Kaplan-Meier survival curves (right) of mice treated with ABX002 surrogate, anti-NKG2A antibody, or isotype control (*n* = 17 per group). The number of surviving mice at the study endpoint is indicated in matching colors on the corresponding survival graph. (D) Tumor growth comparison between mice previously treated with the ABX002 surrogate and rechallenged with A20 tumor cells (*n* = 6), versus naive mice engrafted with A20 tumor cells (*n* = 5). Triangle markers indicate the day of tumor engraftment. (E) Mean tumor volume (left) and survival curves (right) comparing wild-type mice treated with isotype control or ABX002 surrogate versus CD8^+^ T cell-depleted mice treated with ABX002 surrogate (*n* = 10 per group). (F) Mean tumor volume (left) and survival curves (right) comparing wild-type mice treated with isotype control or ABX002 surrogate versus NK cell-depleted mice treated with ABX002 surrogate (*n* = 10 per group). (G) Flow cytometric analysis of A20 tumor samples stained *ex vivo* from mice treated with isotype control antibody. Staining was performed using ABX002 surrogate (blue) or anti-Qa-1^b^ (clone 6A8; pink) to assess expression of Qdm/Qa-1^b^ complexes. gMFI values were background-subtracted by removing signal from cells stained with isotype control. Error bars represent mean ± SD. Data represent combined results from at least two independent experiments. Statistical significance was determined using one-way ANOVA (E, F) or the Mantel Cox log rank test (C, E, F). Significance thresholds: *p* < 0.05 (∗), *p* < 0.01 (∗∗), *p* < 0.001 (∗∗∗), *p* < 0.0001 (∗∗∗∗).

To translate our findings with ABX002 to *in vivo* models, we utilized EXX-1, an ABX002 surrogate and a TCRm antibody specific for the fSP/HLA-E found in mice, Qdm/Qa-1^b^ and previously developed by our team.^30^ For this purpose, BALB/c mice were engrafted with A20 lymphoma cells and treated every other day starting on day 9 and ending on day 21 with two formats of the ABX002 surrogate, anti-NKG2A or control antibody (Figure 5A). The two ABX002 surrogate variants included: (1) one with a standard mouse IgG2A and (2) another having LALA mutations introduced into the Fc domain (ES) to eliminate FcR-driven immune activation.

Treatment with the ABX002 surrogate (ES) led to significant tumor regression compared to the isotype control treated cohort, demonstrating its efficacy in disrupting NKG2A-mediated immune suppression *in vivo*. Moreover, the use of ABX002 surrogate containing an active Fc domain to potentially activate FcR-mediated effector functions was found to further enhance tumor regression (Figure 5B). These findings provide additional support for using ABX002 with an Fc-active domain for immune checkpoint blockade to recruit and activate Fcγ receptor expressing effector cells to enhance anti-tumor responses.

Based on the superior *in vivo* efficacy results observed for ABX002 surrogate with Fc-active domain compared to the effector-silent (ES) variant, we next sought to evaluate the *in vivo* therapeutic potential of ABX002 surrogate having an active Fc domain compared to treatment with anti-NKG2A antibody (clone 20D5).^45^ As expected, monotherapy with anti-NKG2A antibody conferred only modest effect with an 11% survival rate. In contrast, ABX002 surrogate led to significant tumor regression and had a 35% survival rate (Figure 5C). To determine whether treatment with the ABX002 surrogate resulted in durable immunity, we rechallenged animals that had previously achieved complete responses following ABX002 surrogate monotherapy with the same tumor cell line. Remarkably, none of the rechallenged mice developed tumors, indicating the establishment of durable immunity likely due to generation of tumor-specific CD8^+^ T cells resulting from receiving prior treatment with ABX002 surrogate (Figure 5D).

To further dissect the cellular mechanisms underlying anti-tumor immunity, immune cell depletion studies were performed in BALB/c mice. Mice were depleted of either CD8^+^ T cells or NK cells prior to subcutaneous implantation of A20 tumor cells and treatment with the ABX002 surrogate. In both CD8^+^ T cell depleted (Figure 5E) and NK cell depleted (Figure 5F) cohorts, the therapeutic effect of the ABX002 surrogate was largely abrogated, demonstrating that both NK cells (up to the end of week 2) and CD8^+^ T cells (up to the end of week 3) are required for tumor control. Successful depletion of the respective effector cell populations was confirmed by whole-blood staining (Figure S5). Finally to confirm that tumors in mice expressed and the Qdm/Qa-1^b^ complex, we performed *ex vivo* staining of tumor cell samples harvested from control-treated mice. Tumor cells were stained with either ABX002 surrogate (ES), anti-Qa-1^b^ (clone 6A8), or their respective isotype controls. Our analysis confirmed the presence of the Qdm/Qa-1^b^ complex on tumor cells, supporting the functional relevance *in vivo* of these ligands on the surface of tumor cells (Figure 5G).

Together, these studies demonstrate that both NK cells and CD8^+^ T cells are required for the full antitumor activity of the ABX002 surrogate. Even though the relative and temporal contributions of these immune subsets cannot be definitively resolved from these experiments alone and will require longitudinal analyses of tumor-infiltrating lymphocytes in future studies, these findings support the conclusion that MHC-E:NKG2A blockade on both NK cells and CD8^+^ T cells contributes to effective tumor regression and durable anti-tumor immunity.

### Combination therapy using ABX002 surrogate enhances efficacy of PD1/PD-L1 blockade

**Figure 6:**
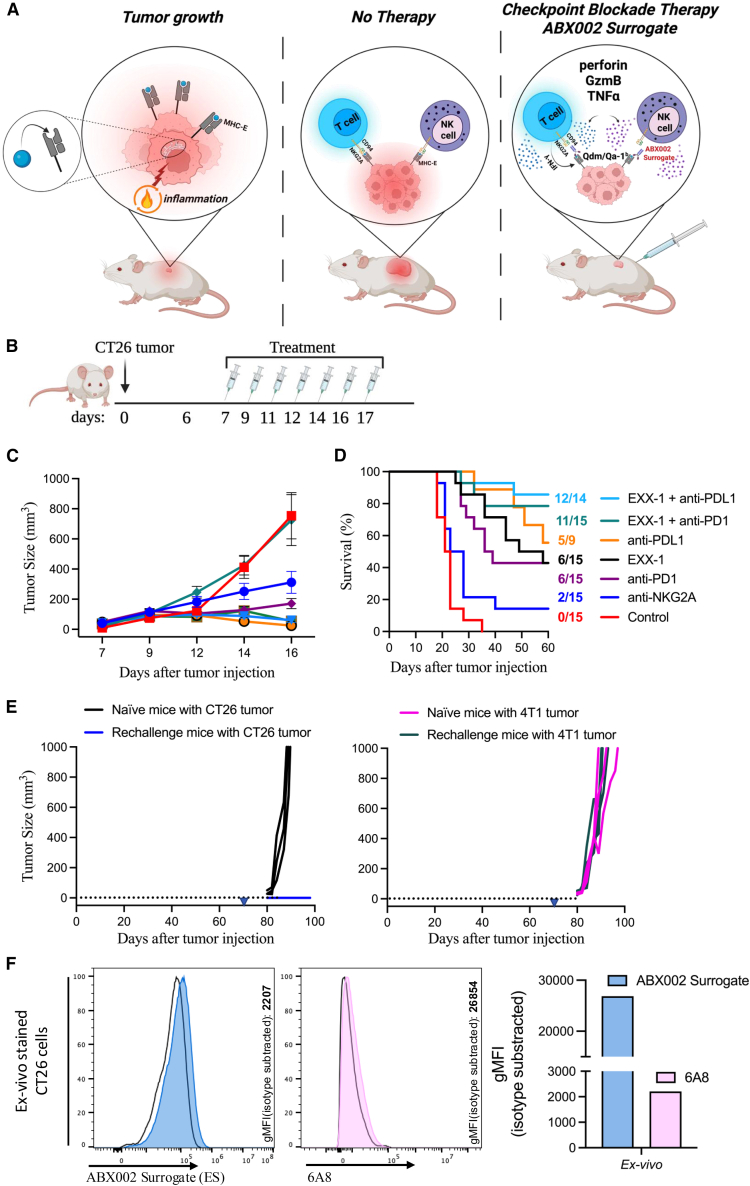
Combination therapy with ABX002 surrogate and anti-PD-(L)1 antibodies enhances therapeutic efficacy in a mouse colorectal tumor model (A) Schematic representation illustrating that MHC class I SPs required for CD94/NKG2A engagement are presented on the cell surface only following inflammatory stimulation. (B) Experimental schematic of the CT26 tumor model and treatment strategy. (C) Mean tumor growth curves in mice treated with monotherapy (ABX002 surrogate or anti-PD-L1) or combination therapy. (D) Kaplan-Meier survival curves for each treatment group, with the number of surviving mice indicated alongside the survival plot (*n* = 15 per group; anti PD-L1 group *n* = 9). Individual tumor growth curves for each group are shown in Figure S6. (E) Tumor growth comparison between mice previously treated with the ABX002 surrogate and rechallenged with CT26 or 4T1 tumor cells (*n* = 3), versus naive mice newly engrafted with CT26 or 4T1 tumor cells (*n* = 4). Triangle markers denote the day of tumor engraftment. (F) Flow cytometric analysis of tumor samples from control-treated mice. Tumors were stained *ex vivo* using ABX002 surrogate (blue) and anti Qa-1^b^ (clone 6A8; pink) to detect Qa-1^b^ heavy chain and Qdm/Qa-1^b^ complexes, respectively. Background-subtracted gMFI values were calculated by removing signal from isotype control stained samples. Error bars represent mean ± SD. See also Figure S6.

To further investigate the therapeutic potential of the ABX002 surrogate, we employed the CT26 colon carcinoma cell line as an additional tumor model in Balb/c mice. CT26 cancer cells represent an immunologically hot tumor characterized by the infiltration of effector immune cells, particularly CD8^+^ T cells, into the tumor microenvironment (TME). The trafficking of these immune cells leads to the local production of inflammatory cytokines, such as IFN-γ, which induces the expression of PD-L1 by tumor cells as a mechanism of adaptive immune resistance. This makes CT26 tumors responsive to PD-(L)1 therapy.^46^^,^^47^ On the other hand, IFN-γ-rich TMEs upregulate the processing and expression of fSP by MHC-E, providing an additional escape mechanism for tumor cells through activating the fSP/HLA-E: NKG2A inhibitory pathway. This model is thus well-suited for assessing combinatorial checkpoint blockade strategies (Figure 6A).

Based on this rationale, we decided to compare the therapeutic effects of the ABX002 surrogate with anti-PD-(L)1 and anti-mouse NKG2A monotherapies, as well as the potential benefits of combination strategies involving the ABX002 surrogate and PD-(L)1 blockade. Mice were subcutaneously engrafted with CT26 cells and were then treated from days 7 to 17 post-engraftment for a total of 7 doses (Figure 6B). Some cohorts received treatments with a combination of ABX002 surrogate and either PD-1 or PD-L1 blockade while other groups received single-agent treatments with either ABX002 surrogate, anti-PD-(L)1, anti-NKG2A, or control antibodies with the objective to assess potential synergistic effects.

The findings from this experiment showed that anti-NKG2A monotherapy rescued only 13% of tumor-bearing mice. By contrast, single-agent treatments with either ABX002 surrogate, anti-PD1, or anti-PD-L1 rescued 40%–55% of mice from death compared to the control-treated group. Notably, the combination therapy groups exhibited a pronounced synergistic effect, rescuing 73%–85% of treated mice when the ABX002 surrogate was paired with either PD-1 or PD-L1 antibody blockade, respectively. Accordingly the combination therapies demonstrated enhanced tumor regression and improved survival rates (Figures 6C and 6D). Individual tumor growth for each condition is shown in Figure S6.

To assess the durability of the immune responses elicited by ABX002 surrogate treatment, we performed a rechallenge experiment on mice that had previously achieved complete responses following monotherapy. Remarkably, none of the rechallenged mice developed tumors, indicating the establishment of durable immunity likely due to the generation of tumor-specific CD8^+^ T cells from prior treatment. Conversely, when these mice were engrafted with a different tumor (4T1), they failed to reject it, confirming that anti-tumor immunity was indeed tumor specific and initiated by ABX002 surrogate therapy (Figure 6E).

Finally, similar to our findings in the A20 tumor model, we verified that the Qdm/Qa-1^b^ complex was expressed on the surface of CT26 tumor cells. Harvested tumors from control-treated mice were stained *ex vivo* with either ABX002 surrogate, anti-Qa-1^b^, or their respective isotype controls. Our analysis confirmed the presence of the Qdm/Qa-1^b^ complex on tumor cells, supporting the functional relevance of these ligands in the observed therapeutic outcomes (Figure 6F). These findings underscore the therapeutic potential of targeting fSP/MHC-E complexes compared to their receptor counterparts to overcome NKG2A-mediated immune inhibition and promote effector cell-mediated anti-tumor activity.

## Discussion

We describe the development and characterization of ABX002, a fully human and first-in-class TCR-mimic antibody that selectively recognizes all tested SPs (VL9) presented by HLA-E∗01:01 and HLA-E∗01:03. ABX002 binds endogenously processed SP/HLA-E complexes with high affinity, shows strict peptide dependence, and exhibits no detectable cross-reactivity with other HLA class I alleles or with non-SPs. Importantly, ABX002 also recognizes rhesus macaque MAMU-E presenting VL9 peptides, establishing translational relevance for preclinical safety and toxicology studies. Together, these properties distinguish ABX002 as both a mechanistic probe for dissecting HLA-E biology and a therapeutic lead capable of targeting tumor cells that elevate VL9/HLA-E to engage the CD94/NKG2A inhibitory checkpoint.

Although HLA-E transcripts are widely detected in human tissues,^48^^,^^49^ it surface expression is comparatively low in resting or homeostatic conditions because HLA-E relies on a limited subset of peptide substrates for stabilization and follow peptide loading pathways differ from classical MHC I.^24^ Inflammatory cytokines such as IFN-γ and α drive HLA-E upregulate HLA-E synthesis and enhance the availability of SPs that can occupy the its binding groove, thereby promoting engagement of the inhibitory NKG2A receptor.^25^^,^^30^^,^^50^^,^^51^^,^^52^ Our results using ABX002 reinforce this regulatory structure. Under basal conditions, human tumor cell lines expressed readily detectable HLA-E protein based on 3D12 staining but showed minimal canonical VL9/HLA-E, as evidenced by absent ABX002 binding. IFN-γ stimulation produced coordinated increases in total HLA-E and fSP loading, demonstrating that inflammation not only enhances HLA-E transcription and translation but also drives peptide availability and loading. Moreover, TAP1-deficient JEG3 cells lacked ABX002 staining, consistent with the requirement of TAP1 for VL9 transport and presentation. Collectively, these findings show that ABX002 reliably reports on the integrity of the PLC and can be used to interrogate enzymes involved in SP trimming and processing.

Previous antibodies, including the TCRm 3H4, recognized only single fSP/HLA-E variants,^29^^,^^43^^,^^53^ limiting their scope for both mechanistic and therapeutic use. ABX002 overcomes this constraint through broad recognition of all canonical fSP variants, enabling a comprehensive and allele-independent strategy for targeting HLA-E restricted antigens. This ligand-centric approach contrasts with receptor-directed therapeutics such as monalizumab,^40^ whose efficacy depends on the patient’s HLA class I genotype and peptide repertoire.^27^^,^^29^ Consistent with this, blocking fSP/HLA-E (Qdm/Qa-1^b^) in mice produced stronger anti-tumor effects than direct NKG2A inhibition, likely enhanced by Fc-mediated antibody-dependent cellular cytotoxicity (ADCC).^54^^,^^55^^,^^56^ ABX002 now offers a direct means to evaluate these contributions and provides a versatile platform suitable for antibody drug conjugates, T cell engagers, and Fc-enhanced derivatives intended to exploit innate and adaptive immune mechanisms.

Tumor profiling using ABX002 revealed minimal fSP/HLA-E display under basal conditions despite high total HLA-E expression. The induction of fSP/HLA-E following IFN-γ exposure suggests that tumors can transition into a state that strongly engages NKG2A in the presence of inflammatory signals.^51^^,^^52^ In contrast to tumors, most human immune subsets lacked ABX002 reactivity even after IFN-γ stimulation, indicating inefficient processing or loading of canonical SPs. Only monocytes consistently displayed canonical VL9/HLA-E, consistent with previous analysis using an ABX002 surrogate antibody. This selective fSP presentation suggests unique peptide-processing capacities in monocytes, potentially reflecting their high expression of peptide-loading machinery or their specialized role in cross-presentation pathways.

The limited ABX002 staining across immune subsets raises several implications for immune regulation. First, HLA-E expressed on many immune cells may not present canonical VL9 but instead may display noncanonical peptides or exist in peptide-deficient conformations. These alternative states may shift receptor engagement away from NKG2A/NKG2C toward receptors such as LILRB1/2, Stablin1/2, or VISTA.^25^^,^^57^^,^^58^ Indeed, emerging evidence suggests that peptide-deficient or noncanonical HLA-E can serve as a ligand for these receptors and thereby modulate immune activation and tolerance. Supporting this idea, studies using ABX002 surrogate in Qdm-deficient mice showed that alternative leader peptides can occupy Qa-1^b^ and alter immune regulation.^59^^,^^60^ Our findings now provide a human-specific reagent with which the field can systematically map canonical versus noncanonical HLA-E conformers and their associated receptor interactions.

In summary, ABX002 represents both a mechanistic and translational advance a ligand-centric checkpoint inhibitor that bypasses the genotypic and contextual constraints of receptor-directed therapies. Beyond its therapeutic potential, ABX002 enables detailed dissection of how inflammation, peptide loading, and antigen-processing pathways shape HLA-E dynamics and downstream immune interactions. By illuminating the diversity of peptides presented by HLA-E and revealing fundamental differences in HLA-E processing between tumors and immune subsets, ABX002 expands our understanding of nonclassical MHC regulation and identifies new avenues for immunotherapeutic design.

### Limitations of the study

In this study, we did not investigate the effects of fSP/HLA-E blockade on the activating CD94/NKG2C receptor, which can shape NK-cell memory-like responses and may become particularly relevant in contexts of CMV-driven NK-cell expansion. Dissecting how ABX002 influences the balance between inhibitory and activating CD94/NKG2 receptors will be important for predicting therapeutic outcomes. In addition, our *in vivo* work relied on heterotopic tumor models, which may not capture the complexity of tissue-specific microenvironments or the contributions of γδ T cells versus αβ T cells. Future studies employing orthotopic and immune-competent models will be necessary to determine how tissue context and local inflammation influence the distribution and function of canonical versus noncanonical HLA-E species.

## Resource availability

### Lead contact

Further information and requests for resources or reagents should be directed to and will be fulfilled up to reasonable request by the lead contact, Jon A. Weidanz (weidanz@uta.edu).

### Materials availability

ABX002 is now a proprietary therapeutic antibody acquired by Boehringer Ingelheim International GmbH and is not publicly available. Access to the ABX002 surrogate antibody may be subject to a material transfer agreement (MTA).

### Data and code availability

This paper dose not report original code.

Any additional information required to reanalyze the data reported in this paper is available from the lead contact upon request.

## Acknowledgments

We thank Dr. Knut Elbers and Dr. Paul Adam for thoughtful discussions regarding the data.

## Author contributions

Conceptualization, S. Ghaffari and J.A.W.; methodology, S. Ghaffari, K.U.-A., D.W., T.v.H., and J.A.W.; investigation, S. Ghaffari, K.U.-A., G.O., T.T., S. Gimlin, D.W., and J.M.; visualization, S. Ghaffari, K.U.-A., and T.T.; funding acquisition, J.A.W. and D.W.; project administration, S. Ghaffari and J.A.W.; supervision, J.A.W. and T.v.H.; writing – original draft, S. Ghaffari; writing – review and editing, S. Ghaffari, K.U.-A., T.v.H., and J.A.W.

## Declaration of interests

D.W. and J.W. were co-founders, D.W. served as CEO, and J.W. served as chief scientist at Abexxa Biologics, Inc., during the study and had ownership interest (including stock and patents) of Abexxa. J.W. is a consultant for Boehringer-Ingelheim International GmbH. T.v.H. reports receiving a commercial grant from Abexxa and was an advisory board member for the same. J.W. and K.U.-A. are listed as inventors on several issued and published U.S. patents and patent applications related to the antibody platform and methods described in this study, including US20210253713A, US10,981,997 B2, US11,359,023 B2, and US11,976,120 B2. These intellectual property rights are assigned to Boehringer Ingelheim International GmbH. The authors declare that these patents are directly relevant to the subject matter of this manuscript. The status of international filings, including any European applications, is not known to the authors at the time of submission.

## Declaration of generative AI and AI-assisted technologies in the writing process

During the preparation of this work, the author(s) did not use generative AI.

## STAR★Methods

### Key resources table

**Table:** 

REAGENT or RESOURCE	SOURCE	IDENTIFIER
**Antibodies**		

CD3_FITC	BioLegend	Cat# 100204; RRID:AB_312661
CD3_PerCP-Cy5.5	BioLegend	Cat# 100267; RRID:AB_2876392
CD3_APC/Fire810	Cytek Biosciences	Cat# 65-0032; RRID:AB_3064875
CD4_BUV496	BD Biosciences	Cat# 569180; RRID:AB_3684846
CD4_AF700	BioLegend	Cat# 100536; RRID:AB_493701
CD8a_BUV395	BD Biosciences	Cat# 563786; RRID:AB_2732919
CD8a_Violet Fluor450	Tonbo Biosciences	Cat# 75-0081; RRID:AB_2621931
NKp46_PE-Cy7	BioLegend	Cat# 137618; RRID:AB_11219186
NKp46_BV785	BioLegend	Cat# 137637; RRID:AB_2734201
Anti-mouse Qa-1b_PE	BD Biosciences	Cat# 566641; RRID:AB_2869802
CD45_BV570	BioLegend	Cat# 103136; RRID:AB_2562612
Mouse Fc gamma RI/CD64	R and D Systems	Cat# MAB20741; RRID:AB_2246880
CD16/32	Tonbo Biosciences	Cat# 40-0161; RRID:AB_2621443
ABX002 surrogate (EXX-1)_AF64	Produced in house	N/A
ABX002 surrogate (EXX-1)	Produced in house	N/A
ABX002 surrogate (EXX-1) Effector Silenced (ES)	Produced in house	N/A
Mouse IgG2A isotype control	Produced in house	N/A
Mouse IgG2A isotype control_AF647	Produced in house	N/A
Mouse IgG2A Effector silenced	Produced in house	N/A
Goat anti human_APC	Jackson ImmunoResearch Labs	Cat# 109-135-098; RRID:AB_2337690
Goat anti human_APC	Jackson ImmunoResearch Labs	Cat# 115-135-205; RRID:AB_2338642
InVivoPlus anti-mouse CD8a	Bio X Cell	Cat# BE0117; RRID:AB_10950145
InVivoPlus anti-mouse PD-L1	Bio X Cell	Cat# BE0101; RRID:AB_10949073
InVivoMAb rat IgG2b isotype control	Bio X Cell	Cat# BE0090; RRID:AB_1107780
InVivoPlus anti-mouse PD-1	Bio X Cell	Cat# BE0146; RRID:AB_10949053
InVivoMAb anti-mouse NKG2A/C/E	Bio X Cell	Cat# BE0321; RRID:AB_2819048
InVivoMAb rat IgG2a isotype control	Bio X Cell	Cat# BE0089; RRID:AB_1107769
Ultra-LEAF™ Purified anti-Asialo-GM1	BioLegend	Cat# 146002; RRID:AB_2562206
Human NKG2A/CD159a_PE	R and D Systems	Cat# FAB1059P; RRID:AB_2132978
Human NKG2A/CD159a_PE	Beckman Coulter	Cat# IM3291U; RRID:AB_10643228
Human NKG2A/CD159a	Beckman Coulter	Cat# IM2750; RRID:AB_131495
Anti-human CD107a (LAMP-1)	BioLegend	Cat# 328602; RRID:AB_1134259
Anti-human CD14_FITC	BioLegend	Cat# 982502; RRID:AB_2616906
Anti-human CD16_PacificBlue	BioLegend	Cat# 980106; RRID:AB_2650644
Anti-human CD20_BV421	BioLegend	Cat# 302330; RRID:AB_10965543
Anti-human CD3_FITC	BioLegend	Cat# 317306; RRID:AB_571907
Anti-human CD3_APC-Cy7	BioLegend	Cat# 317342; RRID:AB_2563410
Anti-human CD4_PE	BioLegend	Cat# 317410; RRID:AB_571955
Anti-human CD56 (NCAM)_AF700	BioLegend	Cat# 362522; RRID:AB_2564099
Anti-human CD56 (NCAM)_PE-CF594	BD Biosciences	Cat# 562289; RRID:AB_11152080
Anti-human CD8_BV605	BioLegend	Cat# 301040; RRID:AB_2563185
Anti-human CD8_APC	BioLegend	Cat# 301049; RRID:AB_2562054
Anti-human HLA-E_PE	BioLegend	Cat# 342604; RRID:AB_1659249
Peroxidase-AffiniPure Goat Anti-Mouse IgG (H + L)	Jackson ImmunoResearch Labs	Cat# 115-035-003; RRID:AB_10015289
Peroxidase-AffiniPure Goat Anti-Human IgG (H+L)	ImmunoResearch Labs	Cat# 109-035-088; RRID:AB_2337584

**Bacterial and virus strains**		

BL21 E. Coli	NEB	Cat#C2527
10-beta E. coli	NEB	Cat#C3019

**Biological samples**		

Human Peripheral Blood Mononuclear Cells, Frozen	Stemcell	Cat#70025
Human Peripheral Blood Leukopak, Fresh	Stemcell	Cat#70500
Whole Blood	Carter Bloodcare	N/A

**Chemicals, peptides, and recombinant proteins**		

Amplification anti-HQ-HRP multimer	Roche	Cat#760-4602
Anti Flag Tag_AF488	R and D Systems	Cat#IC8529G
Bovine Serum Albumin	Sigma-Aldrich	Cat#A3912
EDTA	Invitrogen	Cat#15575020
Geneticin	Gibco	Cat#310131035
Glutathione (reduced)	Sigma-Aldrich	Cat#G4251
Glutathione (oxidized)	Sigma-Aldrich	Cat#G4376
Glycerol	Thermo Scientific	Cat#A16205
HEPES	Gibco	Cat#15630130
Human IL-2 IS	Miltenyi Biotec	Cat#130-097-745
L-arginine	Fisher Scientific	Cat#BP372100
L-glutamine	ATCC	Cat#30-2214
Liberase TL	Sigma-Aldrich	Cat#05401020001
Lympho prep	Stemcell	Cat#18061
Sodium pyruvate solution	Gibco	Cat#11360070
MEM NEAA	Gibco	Cat#11140-035
All Peptides	Genscript	N/A
Puromycin	Gibco	Cat#A1113803
Recombinant human IFN-g	Stemcell	Cat#78141
Streptavidin_PE	Tonbo Biosciences	Cat#50-4317
TMB Substrate Reagent Set	BD Biosciences	Cat#555214
Tween 20	Sigma-Aldrich	Cat#P1379
β-mercaptoethanol	Millipore-Sigma	Cat#63689
All Peptides	Genscript	N/A
NheI	NEB	Cat#R3131L

**Critical commercial assays**		

Anti-Mouse HRP kit	Roche	Cat#760-4310
BirA Biotin Ligase Kit	Avidity	N/A
Click-it sDIBO alkyne kit for antibody labeling (Alexa Fluor 647)	Thermo Fisher	Cat#C20029
Cytotoxicity Detection Kit (LDH)	Sigma-Aldrich	Cat#11644793001
Resonant Sensors Bionetic 96-well plate	Sigma Aldrich	RSNT-RSI96100N6-5
SiteClick antibody azido modification kit	Thermo Fisher	Cat#S20026
Tumor Dissociation Kit, mouse	Milteny Biotec	130-096-730
Ventana Discovery Amp HQ kit	Roche	Cat#760-052
Ventana Discovery OmniMap kit	Roche	Cat#760-159
Zombie Aqua Fixable Viability Kit	BioLegend	Cat#423102

**Experimental models: Cell lines**		

A20-eGFP-Neo/Fluc-Puro	Imanis Life Science	Cat#CL152
CT26	ATCC	Cat#CRL-2638; RIID:CVCL_7254
COLO205	ATCC	Cat#CCL-222 ; RIID:CVCL_0218
EXP293F	Thermo Fisher Scientific	Cat#A14527; RRID:CVCL_D615
HCT-116	ATCC	Cat#CCL-247; RIID:CVCL_0291
JEG-3	Dr. Van Hall	RIID:CVCL_0363
JEG-3-E_KO	Dr.Van Hall	N/A
JEG-3_TAPKO	Dr.Van Hall	N/A
JY	Dr. Van Hall	RIID:CVCL_0108
K562	Dr.Van Hall	RRID:CVCL_0004
K562.HLA-E	Dr. M. Lampen	N/A
NK92	ATCC	CAt#CRL-2407; RRID:CVCL_2142
RPMI-8226	ATCC	Cat#CRM-CCL-155; RIID:CVCL_0014
SU.86.86	ATCC	Cat#CRL-1837; RIID:CVCL_3881
EBY100	ATCC	Cat#MYA-4941

**Experimental models: Organisms/strains**		

BALB/cAnNCrl	Charles River, USA	Cat#028

**Recombinant DNA**		

Plasmid pFUSE-mouse (m)IgG2A-Fc2	Invivogen	Cat: pfuse-mg2afc2
Plasmid pET-21a(+)	Millipore-Sigma	Cat: 69740
pYES3-CT	Thermo Fisher Scientific	Cat: V825320
pHEN1	NovoPro	Ca:V014197

**Software and algorithms**		

BioRender	BioRender	RRID: SCR_018361; http://biorender.com/
FlowJo V.10	Treestar	RRID: SCR_008520; https://www.flowjo.com/solutions/flowjo
GraphPad Prism V.10	GraphPad	RRID: SCR_002798; http://www.graphpad.com/
ResoVu	Resonant Sensors Inc,	https://www.resonantsensors.com/product/

**Other**		

CaptureSelect™ Biotin Anti-IgG-Fc	Thermo Scientific	Cat#7103262100
QIFIKIT®, Series of coated beads	Agilent	Cat#K007811-8
DPBS	Gibco	Cat#14040133
FBS	Cytiva	Cat#SH30406
FluoroFix buffer	Biolegend	Cat#422101
gentleMACS Octo Dissociator	Miltenyi Biotec	Cat#130-134-029
Ghost viability_V450	Tonbo biosciences	Cat#13-0863
Ghost viability_UV450	Tonbo biosciences	Cat#13-0868
Hiload 26/600 Superdex 75	Cytiva	Cat#28989334
Hiload 26/600 Superdex 200	Cytiva	Cat#28989336
Horse Serum	Gibco	Cat#26050088
Resosence Ultra Mab Pro	Resonant Sensors Inc.	https://www.resonantsensors.com/product/
Iscove’s Modified Dulbecco’s Medium (IMDM)	Cytiva	Cat#SH30228
Lympho prep	Stemcell	Cat#18061
Lysis buffer (red blood cells)	Biolegend	Cat#420302
Matrigel	Millipore-Sigma	Cat#CLS354248
McCoy	ATCC	Cat#30-2007
MHC Monomers/Tetramers	In house	N/A
MyeloCult™ H5100	Stemcell	Cat#5150
Normal Rabbit Serum	Abcam	Cat#ab166640
PBS	Fisher Scientific	BP3994
RPMI-1640	Cytiva	Cat#SH30027
Thermo Scientific™ Pierce™ NeutrAvidin™ Coated Plates	Thermo Scientific	Cat#PI15402
Ventana Discovery Ultra Platform	Roche	RRID:SCR_021254

### Experimental model and study participant details

#### Mice

BALB/c mice were purchased from Charles River Laboratories. Mice were housed in individually ventilated cages, under specific pathogen-free conditions and Institutional Animal Care and Use Committee (IACUC) at the University of Texas at Arlington and conducted with guide for the care and use of laboratory animals of national research council. The health status of the animals was monitored over time and all animals were tested negative for agents listed AALAS (American Association for Laboratory Animal Science) guideline. Mice were allowed to acclimate to the housing facility for one week prior to starting experiments. All mice experiments were performed at the animal facility of the University of Texas (protocol#A20.005), Arlington (UTA) United States of America in accordance with the Guide for the Care and Use of Laboratory Animals of the National Research Council.

#### Human subjects

Leukopaks and whole blood from healthy donors were obtained from Stemcell Technologies or Carter BloodCare using Institutional Review Board (IRB) or Research Ethics Committee (REC)-approved consent forms and protocols. Blood samples were diluted in wash buffer (phosphate-buffered saline supplemented with 2% FBS), layered over 15 mL of Lymphoprep™ (Stemcell Technologies) in 50 mL tubes, and centrifuged at 2,200 rpm for 20 minutes with the brake off. The interphase containing PBMCs was carefully collected and washed twice with wash buffer to remove any residual Lymphoprep™, yielding purified PBMCs. Isolated PBMCs were then cultured overnight in the presence or absence of recombinant human interferon-gamma (IFN-γ).

#### Cell lines

A20-eGFP-Neo/Fluc-Puro was purchased from Imanis Life Science. CT26 cell line (RRID:CVCL_7256), SU86.86 (RRID:CVCL_3881), HCT-116 (RRID:CVCL_0291), COLO 205 (RRID:CVCL_0218), RPMI-8226 (RRID:CVCL_0014), NK-92 (RRID:CVCL_2142) were purchased from American Type Culture Collection (ATCC).

The K562-HLA-E (K562-E), K562 (RRID:CVCL_0004) and JEG3 (RRID:CVCL_0363) parental cell lines , JEG3-E KO, JEG3-TAP1 KO, JY(RRID:CVCL_0108), M1-Flu-specific T cells (#1) were kindly provided by Dr. T. Van Hall Laboratory (LUMC, Leiden, the Netherland). FluM1-specific T cells (#2) was purchased from Astarte.

Unless indicated otherwise, all cell lines were cultured in RPMI with 10 % fetal bovine serum (FBS- Cytiva), 2% penicillin/streptomycin (ATCC) and 2mM glutamine (ATCC) at 37 C and 5% CO2. Additionally, A20 cell line were supplemented with 0.05 nM 2-mercaptoethanol (Millipore-Sigma) and 1 mg/mL neomycin (Gibco) and 1 mg/mL puromycin (Thermo Fisher). HCT-116 was cultured in McCoy’s 5a Medium Modified (ATCC) supplemented with 10% FBS (Cytiva), 2% penicillin/streptomycin (ATCC) and 2mM glutamine (ATCC). K562 cells were cultured in IMDM supplemented with 10% FBS (Cytiva), 2% penicillin/streptomycin (ATCC) and 2mM glutamine (ATCC). NK-92 cell line were cultured in MyeloCult™ H5100 (Stemcell), 8% horse serum (Gibco) % penicillin/streptomycin (ATCC) and 2mM glutamine (ATCC) and supplemented with 20 ng/ml IL-2 (Milteny). K562 cells were cultured in Iscove’s modified Dulbecco’s medium (IMDM, Invitrogen) supplemented with 10% FBS (Cytiva), 2% penicillin/streptomycin (ATCC) and 2mM glutamine (ATCC). All cell lines were authenticated by IDEXX bioanalytics using short tandem repeat markers and all cell lines were frequently tested for mycoplasma. For *in vivo* studies, cells were tested for known mouse pathogens using MAP tests.

### Method details

#### Generation of peptides, proteins and antibodies

All peptides used in this study (Table 1) were synthesized by GenScript Biotech (Piscataway, NJ, USA). ABX002 and the isotype control antibody targeting human TNF-α were produced as human IgG1 and Fc-silenced human IgG1-LALA IgG1 (with mutations at Leu234Ala and at Leu235Ala) variants by ATUM (Newark, CA, USA). Similarly, the ABX002 surrogate and its corresponding isotype control, targeting human CD3, were generated as chimeric mouse IgG2a or Fc-silenced mouse IgG2A-LALA antibodies by ATUM.

For antibody production, Expi293F cells (Thermo Fisher) were transfected with vectors encoding ABX002, the ABX002 surrogate, or their respective isotype controls. Five days post-transfection, the supernatant was harvested, and antibodies were purified using protein A/G resin (GenScript) according to the manufacturer’s protocol. The purity of the antibodies was verified by SDS-PAGE and size-exclusion chromatography (SEC) using analytical-grade Superdex 200 columns.

Blocking antibodies targeting mouse PD-1 (clone RMP1-14) and mouse NKG2A (clone 20D5), along with their isotype controls, were obtained from BioXcell.

Peptide-loaded HLA-A2, MHC-E and their tetramers were generated by cloning the extracellular domain of human HLA-E, rhesus MAMU-E, or murine Qa-1^b^, including a C-terminal BirA peptide sequence for biotinylation, along with human β_2_-microglobulin (β_2_M), into pET21(+) vectors. These constructs were transformed into BL21 (DE3) E. coli (New England Biolabs). Recombinant proteins were expressed, purified from inclusion bodies, and subjected to refolding reactions with synthesized peptides (GenScript) to assemble the peptide/HLA-E complex.^61^^,^^62^

The refolded complexes were concentrated and purified by size-exclusion chromatography (SEC) using a Superdex 75 (S75) column. A fraction of the purified monomeric complex was biotinylated using BirA biotin ligase (Avidity) and re-purified using SEC on the S75 column. The efficiency of biotinylation and peptide loading was assessed using a Sciex X500B QTOF mass spectrometer, which confirmed the presence of the heavy chain, β2M, and the bound peptide.

For tetramer formation, biotinylated monomers were incubated with phycoerythrin (PE)-conjugated streptavidin (Cytek Bio), followed by purification using a Superdex 200 (S200) sizing column on an NGC medium-pressure fast chromatography system (Bio-Rad). Both biotinylated and non-biotinylated monomers were used for ABX002 affinity measurements and specificity assays. The final tetramer complexes were employed in tetramer blocking assays.

All mass spectrometry analyses were performed at the Proteomics Core Lab at the University of Texas Southwestern Medical Center.

#### Screening of phage scFv for peptide/HLA-E complexs

A human scFv phage display library comprising 1.42 × 10^9^ unique clones constructed using pHEN1 phage display vector and screened against L-G/HLA-E complex using iterative rounds of biopanning.^63^ To reduce nonspecific binders, an initial depletion and pre-blocking step was performed using 30 μg of a mixture of five control peptide HLA-A∗02:01 complexes (1: SLLMWITQV, 2: YLEPGPVTA, 3: SLFGQRIEV, 4: YMLDLQPET and 5: KLQCVDLHV), followed by enrichment with 50 μg of L-G/HLA-E. This first round resulted in an enrichment factor of 2.73 × 10⁶ (5 × 10¹¹ input phage; 1.83 × 10⁵ output phage). Two additional rounds of biopanning were subsequently performed. The second round yielded an enrichment factor of 4.03 × 10², while the third round incorporated a reduced depletion load (20 μg of the control complex mixture) and resulted in an enrichment factor of 2.31 × 10². In the fourth round, specificity for SP loaded HLA-E was further refined by depleting the library with 50 μg of control peptide (PODXL2)/HLA-E prior to enrichment with 50 μg of L-G/HLA-E, yielding an enrichment factor of 44. Following the fourth round of biopanning, 40 individual phage clones were isolated and sequenced, revealing a single unique scFv sequence.

#### Mutant construction for cycle 2 and cycle 3 and screening of yeast scFv

The scFv clone identified by phage display was subjected to affinity maturation using yeast surface display. The parental scFv sequence was cloned into a modified pYES3/CT yeast display vector for surface expression as an Aga2-fused scFv. All yeast display experiments were performed in the *Saccharomyces cerevisiae* strain EBY100 as previously described.⁶³ A first affinity maturation library (cycle 2) was generated by introducing one to three amino acid substitutions into the CDRH3 region of the parental scFv according to established methods.^43^ The resulting library was transformed into yeast and subjected to fluorescence-activated cell sorting (FACS) using L-G/HLA-E as the binding target.

The scFv sequence selected from cycle 2 was used as the template for a second affinity maturation (cycle 3), in which amino acid substitutions were introduced into the CDRL3 region using an analogous mutagenesis strategy. Yeast libraries were subjected to additional rounds of FACS using L-G/HLA-E for selection. To improve stability, framework modifications were introduced into the affinity-matured scFv. In the VL domain, the CDRL2 sequence was reverted to the germline IGKV1-39 sequence, thereby removing a predicted N-linked glycosylation site. In the VH domain, framework region 1 residues Gln5 and Met18 were reverted to the germline residues Leu5 and Leu18, respectively. For final selection, yeast were incubated with L-G/HLA-E at 10 nM in the presence of parental antibody formatted as human IgG1 (1 μM) for 45 minutes prior to sorting, thereby applying off-rate selection pressure. Selected clones were expanded for downstream characterization.

#### ELISA

Pierce NeutrAvidin Coated High Capacity plates were washed three times with assay buffer (0.1% BSA, 0.05% Tween-20 in PBS). Biotinylated monomers (peptide/MHC-E complexes) were diluted in assay buffer to a final concentration of 0.25 μg/mL, and 50 μL was added to designated wells. Plates were incubated for 1 hour at room temperature (RT) before being washed three times with 200 μL of assay buffer per well, followed by a 5-minute soaking step.

Primary antibodies, including anti-human HLA-E (clone 3D12) and ABX002, were prepared at a final concentration of 0.25 μg/mL in assay buffer. 50 μL of each antibody solution was added to designated wells and incubated for 1 hour at RT. Plates were then washed five times with 200 μL of assay buffer per well.

To detect bound antibodies, anti-human HRP or anti-mouse HRP conjugates (Jackson ImmunoResearch Laboratories) were diluted 1:5000 in assay buffer and added to the designated wells, followed by a 1-hour incubation at RT. After five additional washes, 50 μL of pre-warmed TMB-ELISA substrate solution (BD Biosciences) was added to each well and incubated for 30 minutes at RT. The reaction was stopped by adding 50 μL of 1 M hydrochloric acid, and optical density was measured at 450 nm using either a Synergy 2 multi-mode microplate reader (BioTek Instruments) or a SpectraMax i3X reader (Molecular Devices).

#### Determination of affinity using a label-free bioassay system

The binding affinity of ABX002 was quantified using the ResoSens Ultra Mab-Pro instrument (Resonant Sensors) with data acquisition and statistical analysis performed using Integrated ResoVu software. The dissociation equilibrium constant (Kd) was determined as follows.

CaptureSelect biotin anti-human IgG-Fc (Thermo Fisher Scientific) was diluted in PBS buffer and immobilized onto NeutrAvidin-coated Bionetics label-free microarray plates at a concentration of 5 μg/mL until equilibrium was reached. The plate was then washed three times with PBS buffer (dilution/wash buffer). ABX002, diluted to 5 μg/mL in dilution/wash buffer (0.1% BSA, 0.05% Tween-20 in PBS), was captured by the immobilized anti-human IgG-Fc until binding equilibrium was attained, followed by three additional washes with dilution/wash buffer.

Serial dilutions of the fSP/HLA-E monomer complexes, starting at 40 μg/mL, were added to designated wells and incubated for 30 minutes to assess binding kinetics. After the association phase, unbound fSP/HLA-E complexes were removed, and the wells were refreshed with wash buffer. The plates were then placed in the reader for approximately 30 minutes to monitor the dissociation rate. Binding affinity measurements were analyzed using TraceDrawer kinetic analysis software, enabling precise quantification of the interaction dynamics between ABX002 and fSP/HLA-E monomer complexes.

#### Stimulation of cell lines for flow cytometry experiments

Unless indicated otherwise, cell lines were stimulated for 24 hours days as follows: 200 IU/mL (10 ng/ml) IFN-γ*.*

#### Immunohistochemistry

All H&E and immunohistochemistry staining was done by NovoVita Histopath Laboratory, (Natick, MA). In brief, OCT-embedded frozen human colorectal carcinoma and lung carcinoma tissue blocks purchased from ProteoGenex were sectioned into 5-7 μm-thick slices using a cryostat and mounted onto Superfrost Plus glass slides. All fresh frozen tumor tissue sections were fixed in 10% formalin for 5 minutes and either treated with 100% ethanol for 10 minutes before staining with ABX002/mIgG1 and mIgG1 isotype control antibody or tissue was treated with 100% ethanol for 10 minutes followed by antigen retrieval at 95^o^C for 8 minutes for 3 cycles and then stained with anti-HLA-E (MEM-E/02) and isotype control antibody. Detection of bound ABX002/mIgG1 in tissue was performed following the AmpHQ protocol (Ventana Discovery Amp HQ kit and Amplification anti-HQ-HRP multimer, Venta/Roche) or for anti-HLA-E antibody staining, detection of bound antibody used the Ventana Discovery OmniMap kit with anti-Mouse HRP. All studies were performed on the Ventana Discovery Ultra Platform with digital images generated using an Aperio GT 450 scanner.

For the competition assay, colorectal carcinoma tissue sections were stained for 1hr with ABX002/mIgG1 (5 μg/ml) alone or in combination with 10 μg/ml of soluble L-G/HLA-E and PODXL2/HLA-E complexes to assess binding specificity. Bound antibody was detected following the AmpHQ protocol with images produced by the Aperio GT 450 scanner.

#### Human NK, neutrophil and eosinophil isolation

Primary human NK cells, neutrophils, and eosinophils were purified from Leukopaks or whole blood obtained from STEMCELL Technologies or Carter BloodCare. Cell isolation was performed using the human NK cell, neutrophil, or eosinophil isolation kits (STEMCELL Technologies) according to the manufacturer’s instructions. Purified neutrophils and eosinophils were stained with ABX002/mIgG1 mutations, 3D12, and their respective controls for phenotypic characterization via flow cytometry. Isolated NK cells were used for *in vitro* cytotoxicity assays.

#### NK cell cytotoxicity assay

Freshly isolated NK cells were cultured in complete RPMI medium (RPMI-1640 supplemented with GlutaMAX, 10% FBS, 50 U/mL penicillin, 50 μg/mL streptomycin, 1× MEM non-essential amino acids, 10 mM HEPES, 1 mM sodium pyruvate, and 55 μM 2-mercaptoethanol). JY cells were pulsed with 10 μM L-C3 or PODXL2, or left unpulsed. Primary NK cells (n = 6) were then co-cultured with the stimulated tumor cells at a 2:1 effector-to-target ratio in the presence of ABX002 (ES), anti-NKG2A, or control antibodies at concentrations of 10, 1, and 0.1 μg/mL.

K562-E cells (positively sorted for fSP/HLA-E) or RPMI-8226 cells were harvested prior to co-culture with primary NK cells at a 3:1 ratio (E:T). Cells were plated on ultra-low attachment plates and incubated in the presence of ABX002 (ES), anti-NKG2A, or control antibodies. Assay plates were maintained at 37°C in a humidified incubator with 5% CO₂ for 4 hours (K-562-E and COLO205) or 24 hours (RPMI-8226). Following incubation, 100 μL of cell supernatant was collected for a lactate dehydrogenase (LDH) release assay (Roche). Optical density was measured at 490 nm using a Synergy 2 multi-mode microplate reader (BioTek Instruments).

#### T-cell cytotoxicity assay

JY target cells were stimulated overnight with human IFN-γ and subsequently pulsed with 10 μg/ml M1/Flu peptide and washed three times before incubation with T cells. M1/Flu specific CD8^+^ cytotoxic T cells then were co-cultured with pulsed tumor cells at 1:1 and 3:1 effector-to-target ratio in the presence of ABX002 (ES), anti-NKG2A, control antibodies or no antibody at concentrations of 10 μg/ml. Assay plates were maintained at 37°C in a humidified incubator with 5% CO₂ for 72 hours. Following incubation, 100 μL of cell supernatant was collected for analysis in a lactate dehydrogenase (LDH) release assay (Roche). Optical density was measured at 490 nm using a Synergy 2 multi-mode microplate reader (BioTek Instruments).

COLO205 tumor cells were stimulated overnight with human IFN-γ and subsequently pulsed with 10 μg/ml M1/Flu peptide and washed three times before incubation with T cells. M1/Flu specific cytotoxic T cell then were co-cultured with pulsed tumor cells 3:1 effector-to-target ratio in the absence or presence of 10 μg/mL of the following antibodies, ABX002 (ES), anti-NKG2A, and isotype control. Cells were plated on ultra-low attachment plates and incubated in the presence of ABX002 (ES), anti-NKG2A, or control antibodies. Assay plates were maintained at 37°C in a humidified incubator with 5% CO₂ for 72 hours. Following incubation, 100 μL of cell supernatant was collected for analysis in a lactate dehydrogenase (LDH) release assay. Optical density was measured at 490 nm using a Synergy 2 multi-mode microplate reader (BioTek Instruments).

Specific lysis was calculated according to the manufacturer formula

$$\frac{Test\;release-spontaneous\;release}{maximum\;release-spontaneous\;release}\times 100$$

#### Flow cytometry

Single-cell suspensions were prepared from cultured cell lines or *ex vivo* harvested mouse tumor tissues, including A20 lymphoma and CT26 tumors. Tumors were mechanically dissociated and enzymatically digested with 2.5 mg/mL Liberase using the Miltenyi Gentle MACS Octo Dissociator under cold lysis conditions, following the manufacturer’s instructions. The resulting cell suspensions were passed through a 70 μm cell strainer and plated into V-bottom 96-well plates. Mouse Fc receptors were blocked using rat anti-mouse CD64 (clone 290322) and CD16/CD32 (clone 2.4G2) for 10 minutes at room temperature in staining buffer (PBS + 2 mM EDTA). Human Fc receptors were blocked by incubating with TruStain FcX for 10 minutes at room temperature. Cell viability was assessed using the LIVE/DEAD Fixable Aqua Dead Cell Stain Kit (BioLegend) or the Ghost Dye Violet 450 Fixable Viability Kit (Cytek), applied during or after surface staining.

PE-conjugated L-G/HLA-E or HSP60/HLA-E tetramers were stained for 30 minutes on ice in staining buffer. Additional surface markers, along with viability dyes, were added in staining buffer for 30 minutes at 4°C. For the NK blocking assay, PE conjugated L-G/HLA-E tetramers were preincubated with ABX002 or 3D12 antibodies for 10 minutes on ice before being added to NK cells. Anti-NKG2A (clone Z199) was incubated with NK cells for 10 minutes on ice prior to the addition of L-G/HLA-E tetramers.

For flow cytometry experiments involving direct conjugation, antibodies were conjugated using the SiteClick antibody azide modification kit and subsequently labeled with Alexa Fluor 647 dye via the SiteClick sDIBO alkyne kit, following the manufacturer’s instructions. When using secondary antibodies for labeling, goat anti-human allophycocyanin (APC) antibodies was used.

Peripheral blood samples were collected from mice 14 days after the initial administration of anti-asialo-GM1 and anti-CD8α antibodies (50 μL per mouse). A staining antibody cocktail containing NKp46-PE Cy7 and CD8-V450 was added to each sample, followed by incubation at room temperature for 15 minutes. Red blood cells (RBCs) were lysed by adding 1 mL of 1× FACS Lysing Solution (diluted in water)to each well and incubated for 10 minutes at room temperature in the dark. The samples were then washed with 2 mL of staining buffer to remove residual lysis buffer and prepared for further analysis.

For all blood-derived cell staining experiments, Fcγ receptors were blocked using a CD16/CD32/CD64 antibody cocktail prior to antibody incubation. ABX002 or its surrogate antibody was used in an Fc-silenced IgG format containing LALA mutations to minimize Fcγ receptor mediated interactions.

K562-E cells were pulsed with 50 μg/mL of peptide, including fSP, non-fSP, non-canonical, and pathogen-derived peptides, and incubated at 37°C and 5% CO₂ for 2 hours. Cells were then washed twice with staining buffer and stained with either 1 μg/mL of ABX002 or 3D12 (for binding specificity assays) or a titrationof ABX002 (1- 0.0001 μg/mL) for avidity determination assays. Staining was performed for 30 minutes at 4°C. After washing with 100 -150 μL of FACS buffer, secondary antibody conjugates were added. GAM-Fc/APC was added at a 1:100 dilution in FACS buffer and incubated for 30 minutes at 4°C. All samples were stained with Ghost Dye Violet 450 viability dye and washed twice with staining buffer before fixation with 50-100 μL of FluoroFix buffer (BioLegend) for subsequent analysis.

For yeast staining and selection, the scFv antibody library was cultured overnight in galactose-rich media at 30°C to induce antibody expression on the yeast surface. Yeast cells were resuspended in yeast blocking buffer (5% BSA, 0.05% Tween-20 in PBS) and incubated for 30 minutes at room temperature with rotation. Cells were then incubated with biotinylated antigens, including L-G/HLA-E and irrelevant control antigens, for 30-90 minutes at 4°C, with incubation time adjusted based on antigen concentration. After incubation, yeast cells were washed and resuspended in yeast staining buffer (0.5% BSA, 0.05% Tween-20 in PBS) containing anti-FLAG (DYKDDDDK) antibodies conjugated to FITC and streptavidin (SA)-R-PE. Cells were incubated for 30 minutes at 4°C, washed three times, and resuspended in yeast staining buffer before analysis by flow cytometry.

For Cell surface antigen quantification we used the Agilent Dako QiFi quantification kit and following the manufacturers instructions. Briefly, cells were labeled with the primary antibody (3D12, ABX002 or isotype). After washing, cells, set-up beads and calibration beads were labeled with the fluorescently-labeled secondary antibody (goat-anti-mouse). Cytometer settings were set based on fluorescence of the set-up beads. The calibration beads contain five populations of beads each having a different number of mouse IgG molecules, based on the lot. Together with MFI values, this is used to generate a calibration curve to extrapolate antigen quantification from sample MFI values.

Stained cells were analyzed using an Aurora 5L spectral flow cytometer (Cytek) or a CytoFLEX S V4-B2-Y4-R3 flow cytometer (Beckman Coulter). Flow cytometry data was processed using FlowJo software version 10.10.0 (Tree Star). A comprehensive list of all antibodies used for flow cytometry is provided in the resources table. All cell sorting experiment performed at Dallas Baylor Scott & White Research Institute.

For data analysis, cells were first gated based on forward and side scatter to exclude debris, followed by selection of singlets and live cells. Yeast cells were analyzed similarly, with additional gating for scFv expression and antigen binding. An overview of all the antibodies used for flow cytometry is shown in the resources table.

#### *In vivo* experiments in mouse tumor models

For tumor inoculation, 2.5 × 10⁵ CT26 and 5 × 10⁶ A20 tumor cells were subcutaneously injected into the flank of BALB/c mice. Tumor cells were resuspended in 100 μL of PBS supplemented with 0.1% BSA prior to injection. Tumor growth was monitored three times per week using a digital caliper, and tumor volume was calculated using the formula:

Volume=Length×(Width2)2.

Once tumors became palpable, mice were randomized into treatment groups for immunotherapy. Mice were euthanized when tumor volumes reached 1,000 mm³. For Kaplan-Meier survival analysis, mice were considered deceased when tumor volumes exceeded 1,000 mm³.

In the A20 tumor model, treatment with checkpoint blockade antibodies commenced when tumor volumes reached 150-200 mm³. Antibodies were administered intraperitoneally (i.p.) at a dose of 200 μg per mouse (20 mg/kg, diluted in PBS) on days 9, 11, 13, 15, 17, and 19 post-transplantation.

For NK cell depletion, BALB/c mice received weekly intraperitoneal injections of 100 μL of polyclonal anti-asialo-GM1 antibody. Normal rabbit serum was used as an isotype control.

For CD8⁺ T cell depletion, each mouse received an initial i.p. injection of 400 μg of depleting anti-CD8α monoclonal antibody (mAb), followed by 200 μg for two subsequent weekly injections. A rat IgG2b κ antibody was used as the control. Immune cell depletion protocols were initiated as specified in the corresponding figure legends, and depletion efficiency was assessed by flow cytometry.

In the CT26 tumor model, tumor-bearing BALB/c mice were treated with blocking antibodies, including ABX002 surrogate, anti-NKG2A (clone 20D5), anti-PD-L1 (clone 10F.9G2), and anti-PD-1 (clone RPM1-14), along with their respective isotype controls. Antibodies were administered intraperitoneally at a dose of 200 μg per mouse (20 mg/kg, diluted in PBS) on days 6, 8, 10, 12, 14, 16, and 18 post-tumor injection.

### Quantification and statistical analysis

All mouse experiments were performed with a minimum of three biological replicates. *In vitro* experiments were performed at least twice. Statistical tests are described in the figure legend. Calculations between two groups used an unpaired two-tailed student’s t-test and between more than two groups used an ANOVA with Tukey’s post-hoc test, unless otherwise indicated. Survival analyses were performed using a log rank Mantel-Cox test. GraphPad Prism (V9.2.0) was used for all statistical testing. Data are represented as mean ± SD unless indicated otherwise. Statistical significance is shown as ∗p < 0.05, ∗∗p < 0.01, ∗∗∗p < 0.001 and ∗∗∗∗p < 0.0001.

### Additional resources

There are no additional resources to report for this study.
