# Supplementary material for: A fully human pan VL9 HLA-E TCRm antibody enables functional dissection of HLA-E biology and checkpoint signaling
Source: iScience. 2026 Apr 9;29(5):115669. doi: 10.1016/j.isci.2026.115669 (PMC13129390; doi:10.1016/j.isci.2026.115669)
Supplement: Document S1. Figures S1–S6 [file mmc1.pdf]

iScience, Volume *29*

Supplemental information

A fully human pan VL9 HLA-E TCRm antibody enables functional dissection of HLA-E biology and checkpoint signaling

Soroush Ghaffari, Katherine Upchurch-Ange, Gizem Oter, Trivendra Tripathi, Susanne Gimlin, Debra Wawro Weidanz, Jim Middelburg, Thorbald van Hall, and Jon A. Weidanz

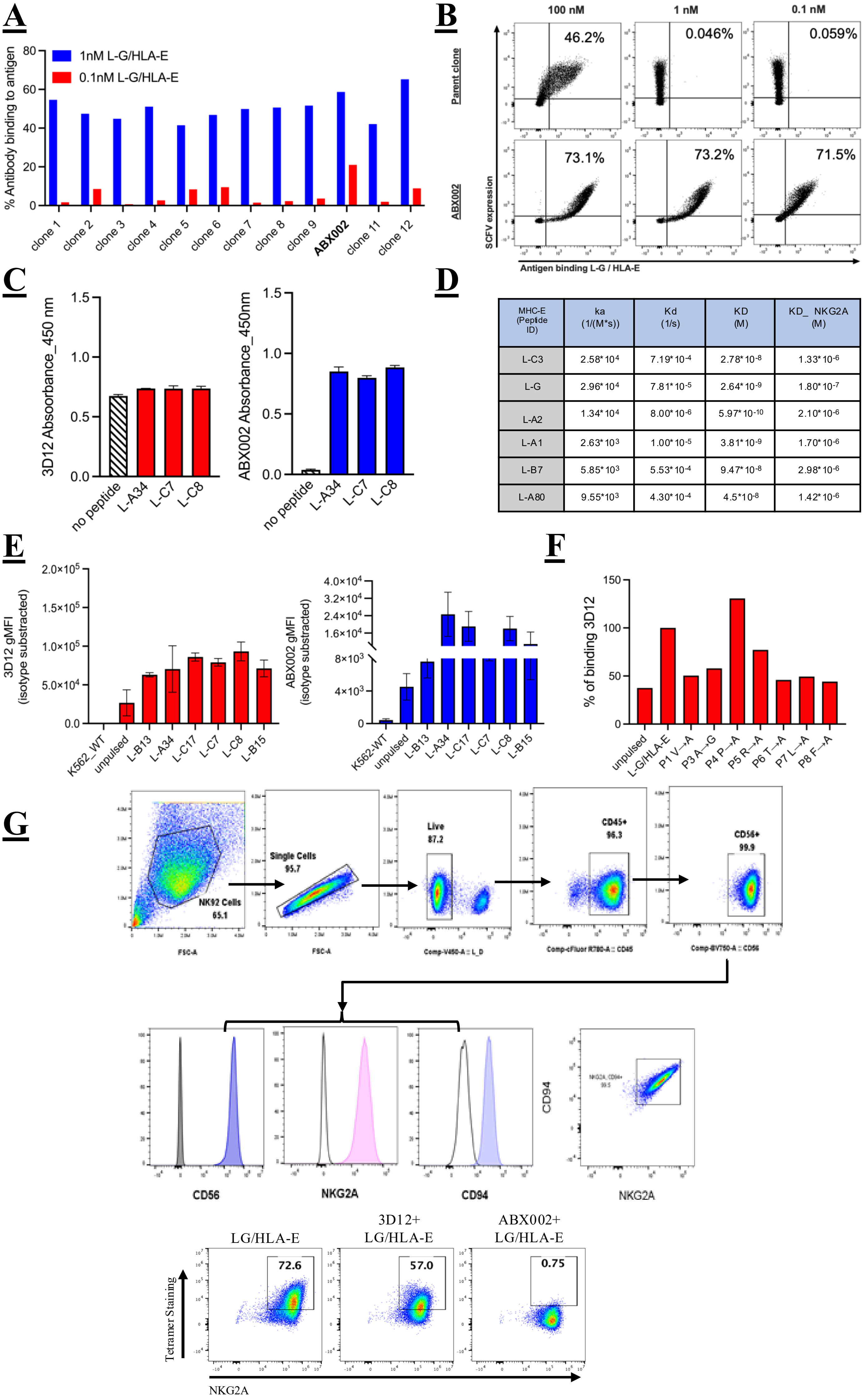

MHC-E (Peptide ID)	ka (1/(M*s))	Kd (1/s)	KD (M)	KD_ NKG2A (M)
L-C3	$2.58*10^{4}$	$7.19*10^{-4}$	$2.78*10^{-8}$	$1.33*10^{-6}$
L-G	$2.96*10^{4}$	$7.81*10^{-5}$	$2.64*10^{-9}$	$1.80*10^{-7}$
L-A2	$1.34*10^{4}$	$8.00*10^{-6}$	$5.97*10^{-10}$	$2.10*10^{-6}$
L-A1	$2.63*10^{3}$	$1.00*10^{-5}$	$3.81*10^{-9}$	$1.70*10^{-6}$
L-B7	$5.85*10^{3}$	$5.53*10^{-4}$	$9.47*10^{-8}$	$2.98*10^{-6}$
L-A80	$9.55*10^{3}$	$4.30*10^{-4}$	$4.5*10^{-8}$	$1.42*10^{-6}$

Fig. S1- Biochemical and functional specificity of ABX002.

Following iterative rounds of selection and affinity maturation, 12 unique scFv clones exhibiting improved binding affinity relative to the parental clone. (**A**) Comparative binding of all 12 clones, including ABX002, to L-G/HLA-E at 1 nM and 0.1 nM antigen concentrations, shown as percent antibody binding to antigen. (**B**) Binding of the parental yeast-displayed scFv (top) and ABX002 (bottom) to L-G/HLA-E at 100 nM, 1 nM, and 0.1 nM. (**C**) ELISA-based assessment of 3D12 (red) and ABX002 (Blue) binding to non-fSP/HLA-E complexes. (**D**) Affinity measurements of ABX002 in comparison with NKG2A ligand. Binding kinetics were determined for all fSP/HLA-E complexes. (**E**) Cell-based analysis of 3D12 (red) and ABX002 (blue) to non-SP peptide-loaded K562-E cells. (**F**) Alanine scanning of the L-G fSP in K562-E cells. 3D12 binding is quantified as gMFI of the indicated peptide normalized to gMFI of L-G × 100. (**G**) Flow cytometry gating strategy used to identify NKG2A^{+} NK92 cells in functional assays.

A

Cell line	HLA type
COLO205	A*01:01,02:01, B*07:02,08:01, C*07:02
JEG-3	C*01:04
JY	A*02:01,02:01, B*07:02,07:02, C*07:02,07:02
K562	A11:01, C5:01
RPMI-8226	A*30:01,68:02, B*15:03,15:10, C* 02:02,03:04,
SNU-899	A*02:06,02:06, B*15:01,15:01, C*04:01,04:01
SU.86.86	A*29:02,32:01, B*44:02,44:02, C*05:01,16:01
HCT-116	A*01:01,02:01, B*45:01, C*05,07

B

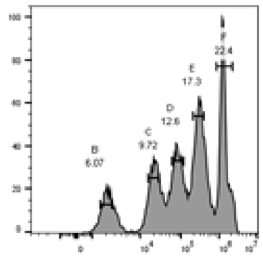

Beads	Aby molc/bead	GeoMean
B	1700	2058
C	10000	19896
D	42000	77403
E	179000	278295
F	759000	1240000

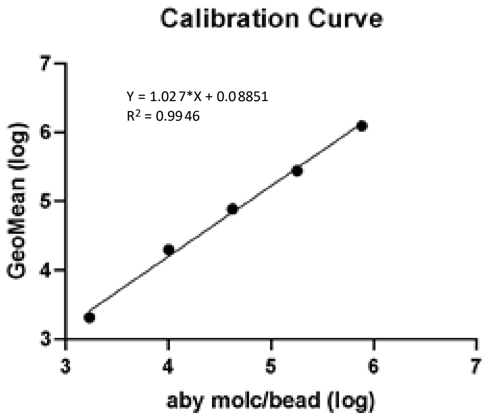

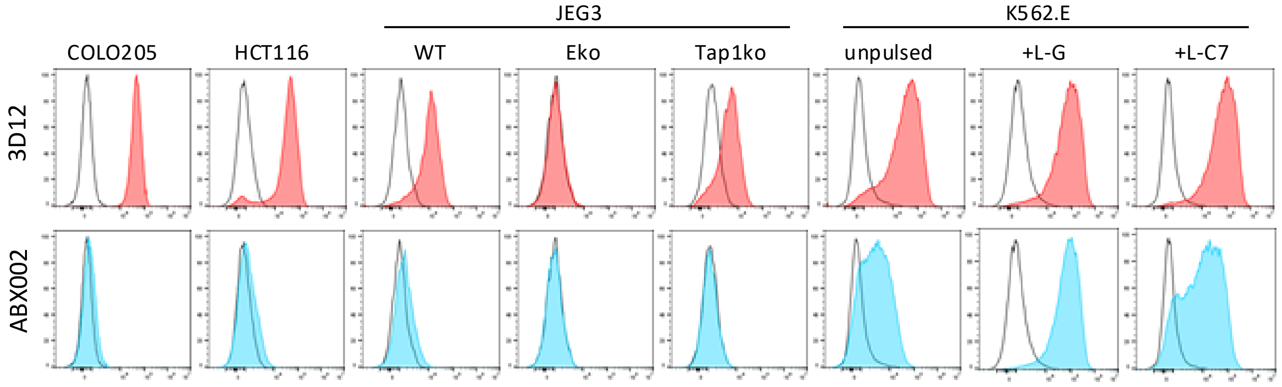

Antibody	COLO205	HCT116	JEG3			K562.E		
			WT	Eko	Tap1ko	unpulsed	+L-G	+L-C7
3D12	24,856	15,184	5,176	45	2,025	14,327	32,871	34,686
ABX002	180	264	355	0	5	1,539	35,160	4,779

Fig. S2. HLA genotype of tumor cells and sensitivity threshold for ABX002 binding to fSP/HLA-E.
(A) HLA class I and HLA-E genotyping of tumor cell lines used in ABX002 binding specificity assays (refer to Figure 2A), including COLO205, HCT116, JEG3 (wild-type and genetically modified), and K562-E. **(B)** Cell surface antigen quantification for ABX002 binding to fSP/HLA-E complexes compared to total HLA-E expression. HLA-E protein levels were measured using the anti-HLA-E monoclonal antibody 3D12, while fSP/HLA-E complex detection was assessed using ABX002.Tumor models included COLO205, HCT116, wild-type and engineered JEG3 cells (after 24 hrs treatment with IFN-γ), as well as K562-E cells with or without peptide pulsing. Data indicate the number of antibodies binding per cell as extrapolated from the standard curve.

A

Donor #	Donor ID	CMV status	HLA type	Functional peptides
1	220581902C	Negative	A02:01, B07:02, B44:02, C05:01, C07:02	L-A2, L-B7,L-B13, L-Cw3, L-Cw7
2	2210404004	Positive	A01:01, A30:02, B14:02, B58:01, C07:01, C08:02	L-A1, L-B7, L-B15, L-Cw3, L-Cw7
3	2304403013	Positive	A02:02, A30:01, B15:10, B42:01, C03:04, C17:01	L-A2, L-A1, L-B15, L-B7, L-Cw3, L-Cw17
4	220281707C	Positive	A03:01, A68:02, B07:02, B81:01, C08:04, C04:01	L-A1, L-A2, L-B7, L-Cw3
5	2306814103	Positive	A02:01, A30:02, B07:02, B27:05, C02:02, C07:02	L-A2, L-A1, L-B7, L-B13, L-Cw7
6	2206401002	Positive	A24:07, A35:05, B35:01, B38:02, C04:01, C07:02	L-A2, L-B15, L-B7, L-Cw3, L-Cw7
7	2201418004	Negative	A03:01, A32:01, B35:01, B35:02, C04:01	L-A1, L-B15, L-Cw3
8	220272101C	Negative	A01:01, A03:01, B08:01, B35:01, C07:01, C04:01	L-A1, L-B7, L-B15, L-Cw7, L-Cw3
9	220182001C	Positive	A02:02, A33:03, B07:02, B39:10, C07:02, C12:03	L-A2, L-A1, L-B7, L-Cw3, L-Cw7
10	230442400	Positive	A03:01, B45:01, B49:01, C07:01, C15:05	L-A1, L-B15, L-Cw7

B

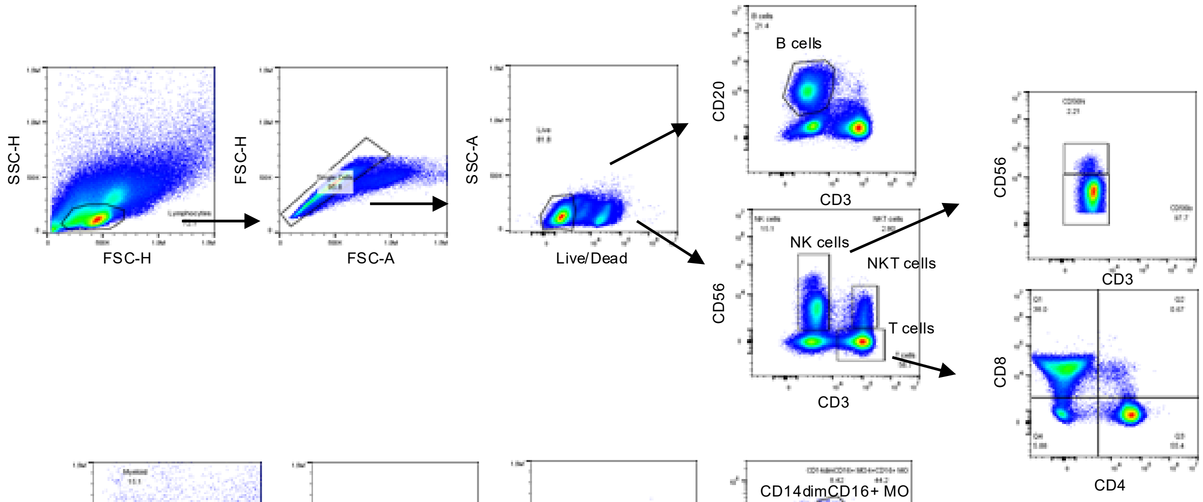

C

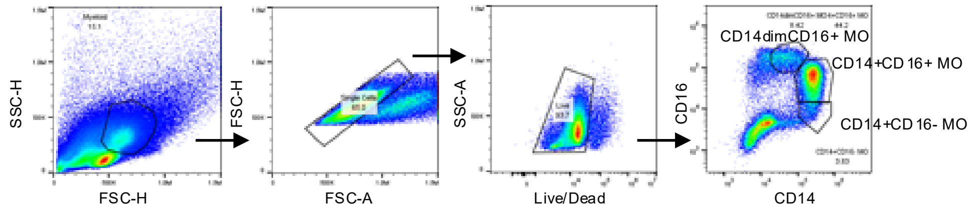

D

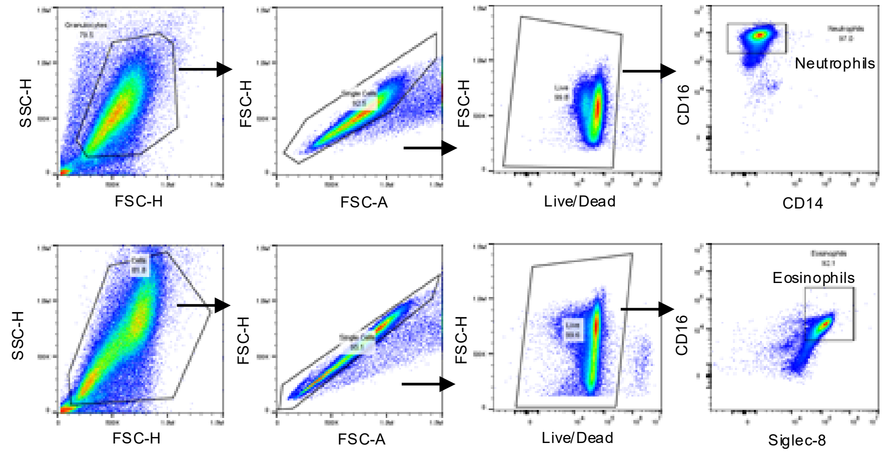

E

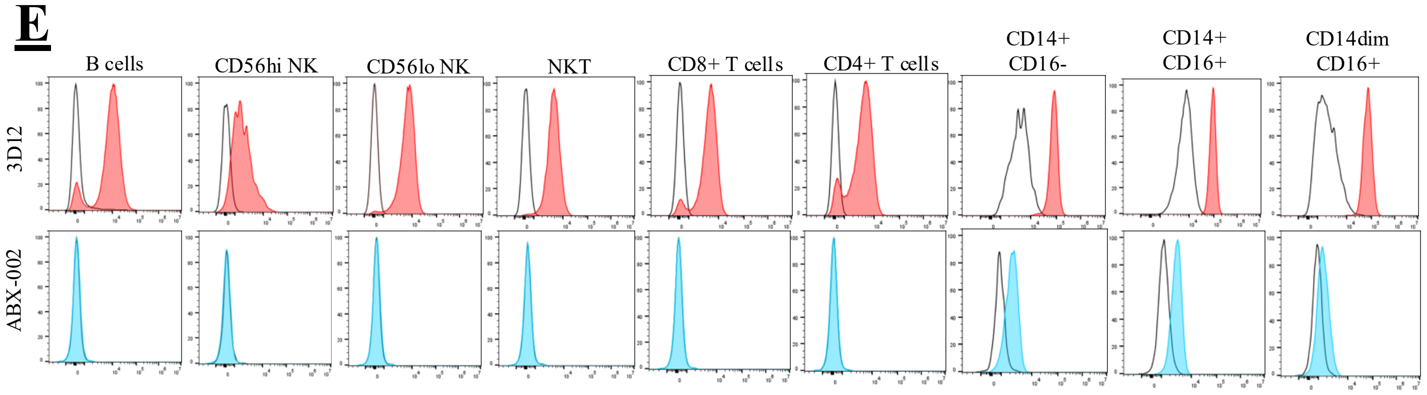

Fig. S3. HLA typing of PBMC donors and gating strategy for immune cell subpopulations.
(A) HLA class I and HLA-E typing of peripheral blood mononuclear cell (PBMC) donors used in the ex vivo binding assays shown in fig 3C. **(B)** Flow cytometry gating strategy for major lymphocyte subsets, including B cells, NK cells ($CD56^+$ subsets: CD56^hi and CD56^low), NKT cells, and $CD4^+$ and $CD8^+$ T cells. **(C)** Gating strategy for identification of monocyte populations. **(D)** Gating strategy for granulocyte subsets, including neutrophils and eosinophils. **(E)** Representative flow cytometric histograms from a single donor illustrating ABX002 and 3D12 staining across immune cell subsets.

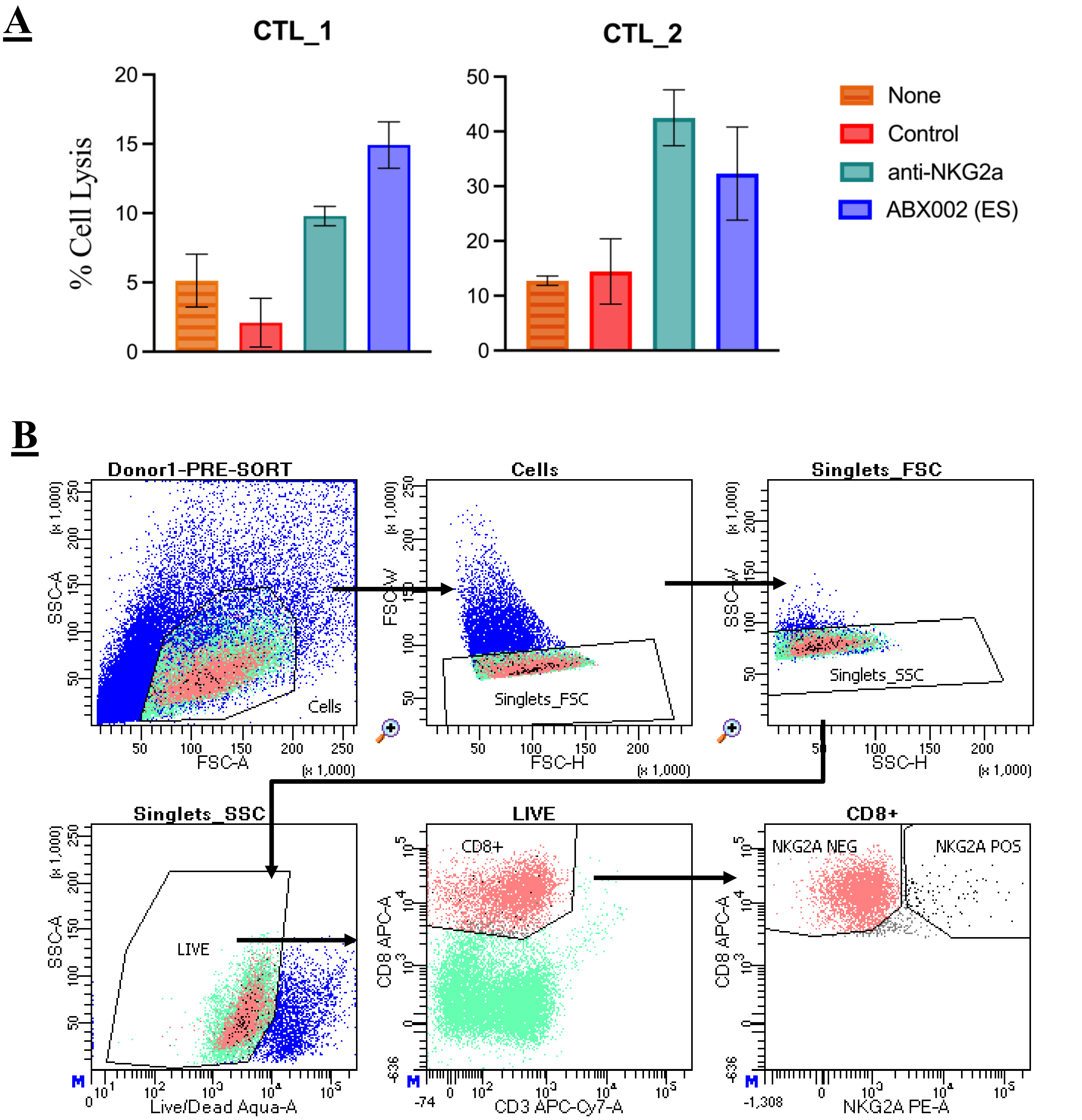

Fig. S4. ABX002 enhances antigen-specific cytotoxic T cell–mediated lysis of COLO205 tumor cells.
(A) Functional killing assay using anti-M1/Flu NKG2A⁺CD8⁺ cytotoxic T lymphocytes (CTLs) derived from two independent donors. CTLs were co-cultured with IFN-γ stimulated COLO205 cells that pulsed with M1/Flu peptide in the presence of ABX002 (effector-silenced format), anti-NKG2A blocking antibody, or respective controls. (**B**) Flow cytometry gating strategy for sorting NKG2A⁺ and NKG2A⁻ CD8⁺ T cells from donors D1 and D2 used in the functional assay depicted in Figure 4C. (**A**) Data are presented as mean ± standard deviation (SD) from technical replicates.

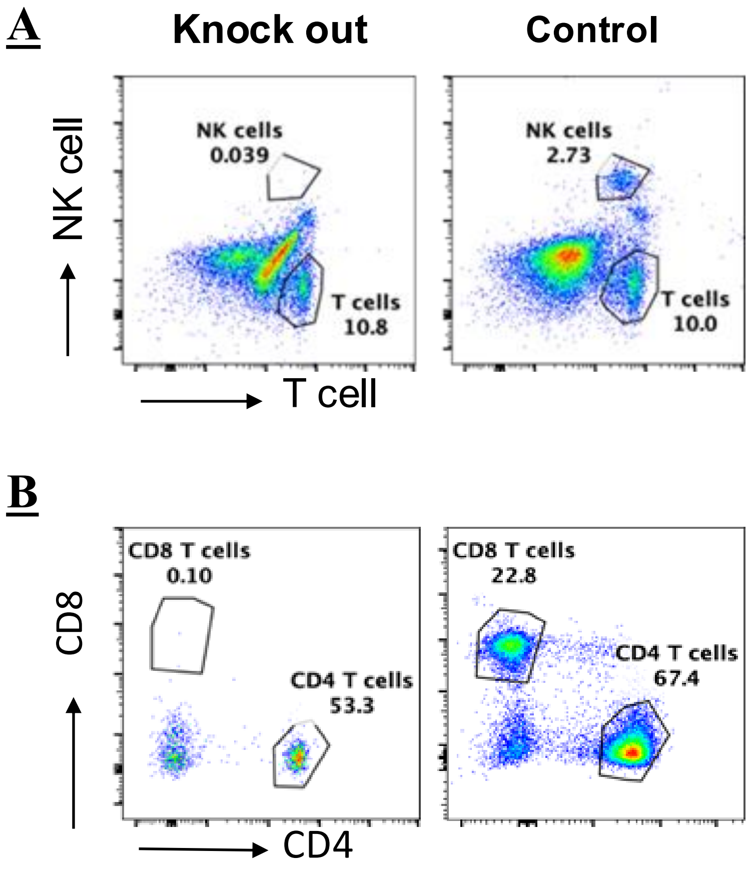

Fig. S5. Flow cytometric analysis of immune cell populations from mice depleted of NK cells or CD8$^+$ T cells. Representative flow cytometry histograms of splenocytes from knockout mice (left) and wild-type control mice (right). (A) NK cell–deficient mice and corresponding wild-type controls. (B) CD8$^+$ T cell–deficient mice and corresponding wild-type controls.

A

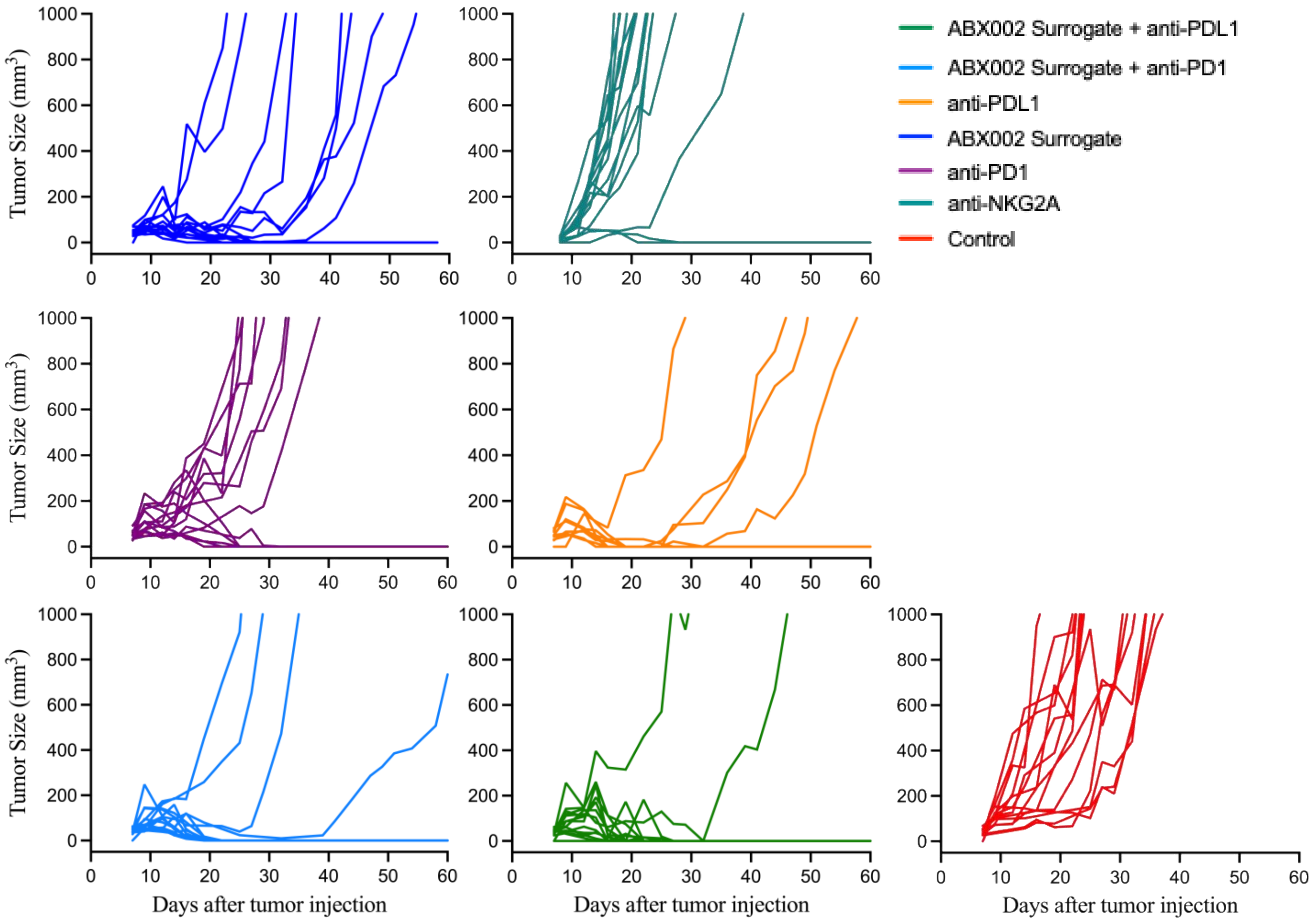

Fig. S6. Individual tumor growth kinetics following single-agent or combinatorial antibody therapy.
Tumor volume measurements in individual CT26 tumor bearing mice treated with indicated antibody regimens. Mice were monitored longitudinally for tumor progression, and individual tumor sizes were recorded over time for each treatment group to illustrate inter-animal variability in response to therapy.
